# Bilingualism and language similarity modify the neural mechanisms of selective attention

**DOI:** 10.1038/s41598-019-44782-3

**Published:** 2019-06-03

**Authors:** Andrea Olguin, Mario Cekic, Tristan A. Bekinschtein, Napoleon Katsos, Mirjana Bozic

**Affiliations:** 1 Department of Psychology, University of Cambridge, Downing Street, Cambridge, CB2 3EB UK; 2 Department of Computer Science and Technology, University of Cambridge, 15 JJ Thomson Ave, Cambridge, CB3 0FD UK; 3 Department of Theoretical and Applied Linguistics, University of Cambridge, Sidgwick Avenue, Cambridge, CB3 9DA UK

**Keywords:** Language, Human behaviour

## Abstract

Learning and using multiple languages places major demands on our neurocognitive system, which can impact the way the brain processes information. Here we investigated how early bilingualism influences the neural mechanisms of auditory selective attention, and whether this is further affected by the typological similarity between languages. We tested the neural encoding of continuous attended speech in early balanced bilinguals of typologically similar (Dutch-English) and dissimilar languages (Spanish-English) and compared them to results from English monolinguals we reported earlier. In a dichotic listening paradigm, participants attended to a narrative in their native language while ignoring different types of interference in the other ear. The results revealed that bilingualism modulates the neural mechanisms of selective attention even in the absence of consistent behavioural differences between monolinguals and bilinguals. They also suggested that typological similarity between languages helps fine-tune this modulation, reflecting life-long experiences with resolving competition between more or less similar candidates. The effects were consistent over the time-course of the narrative and suggest that learning a second language at an early age triggers neuroplastic adaptation of the attentional processing system.

## Introduction

Humans are capable of learning multiple languages without major difficulty, especially at an early age. While this brings obvious advantages such as intercultural communication and enhanced career prospects, bilingualism has also been linked to changes to selective attention and inhibition of unwanted information^1–3^. Although behavioral differences between monolinguals and bilinguals on tasks of selective attention remain controversial^4,5^, the experience of learning and using a second language undoubtedly represents a major environmental demand that can impact the way the brain processes information^6,7^. Here we investigate how early second-language acquisition influences the neural mechanisms of auditory selective attention, and whether this is further affected by the typological similarity between the two languages. We address these questions in a natural listening context, by investigating the neural encoding of continuous attended narratives under different types of linguistic and non-linguistic interference in Spanish-English and Dutch-English early bilinguals.

## Auditory Selective Attention and Bilingualism

Selective attention is the ability to sustain focus on task-relevant stimuli in the presence of distractors. Historically, two major views guiding research on auditory selective attention were the ‘early-selection’ and the ‘late-selection’ approaches^8–10^, where the early-selection theories argued that, due to our limited processing capacity, attended and unattended information is differentiated early on; while late-selection accounts proposed that selective attention dissociates inputs based on semantic encoding and analysis, after both streams had undergone equivalent perceptual processing. Subsequent theories argued that unattended information might be attenuated rather than completely filtered out, allowing some unattended information to reach awareness^11^; that selective attention is flexible, such that attended and unattended items can be discriminated at different depths of analysis^12^; and that early attention relies on basic signal properties (sound level, fundamental frequency), allowing for fast selection, while late attention utilizes syntactic and semantic information and is used for slow selection^13^. Despite their differences, one hypothesis shared by most accounts is that selective attention is a cognitive faculty with limited capacity. The hypothesis that bilingualism can affect these mechanisms stems from the concept of non-selective lexical access as introduced by the BIA framework^14–16^, which is strongly supported by findings that both languages are simultaneously active in the bilingual’s brain, and that bilinguals regularly switch between them and inhibit the unwanted one^17–23^. Additionally, a number of studies reported that the same neural network underpins the processing of both languages^24,25^. This constant need to inhibit the activation of the non-target language within the same network was argued to elicit the enhancement of attentional control and the ability to inhibit unwanted information^26,27^. While many studies reported that bilinguals tend to outperform monolinguals in tasks of attentional control and inhibition^28,29^ (but see^30,31^), there are also questions about the reliability of such findings, or about the specific contexts of bilingual language learning and use that may give rise to such differences^5,32^.

## Neuroplasticity as a Function of L2 Experience

Even if the behavioural findings about enhanced attentional control cannot be generalized across tasks and different types of bilinguals, it is unequivocal that learning and using multiple languages represents a major environmental demand, which can modify the way the brain processes information. This reflects the brain’s capacity to adapt to changes in the environment, and is equivalent to learning-induced neural changes seen across other cognitive domains^33–35^. A number of studies investigated how experience with a second language modifies the underlying neural processing, exploring both anatomical and functional differences between monolinguals and bilinguals^36,37^. Results suggest that bilinguals show increased grey matter density^38–40^ and white matter connectivity compared to monolinguals^41,42^; as well as less activation in structures related to executive control while still outperforming monolinguals^43^, arguably indicating the presence of a more effective control network.

In the auditory domain the evidence is somewhat limited, with some studies focusing on the processing of isolated syllables only. The existing results show stronger subcortical encoding of the fundamental frequency (F0) and more consistent responses to attended syllables in both subcortical and cortical areas in bilinguals^6,44^, as well as an earlier frontal positivity for primed spoken words, indicating enhanced selective attention^45^. A recent study^46^ found that bilingualism can modify the early processing of sound even during pre-attentive listening. Yet, while these studies provide evidence for neural changes in response to the demands of bilingualism, the literature on the relationship between bilingualism and indices of managing interfering information remains inconsistent^47^. In particular, how bilingualism modifies the way speakers track and encode natural continuous speech in the presence of interference remains largely unknown.

## Neural Encoding of Attended and Unattended Speech

The speech signal is strongly encoded in the brain. Studies have shown significant correlations between neural activity and the attended speech envelope^48–50^, with modulations of the speech envelope (corresponding to syllabic or phonetic rate of speech) robustly synchronized to the low-frequency neural oscillations^51,52^. This phenomenon has been referred to as the Selective Entrainment Hypothesis^53–55^. Encoding can also be observed for higher-level lexical information, with the brain responding to the semantic content of words in a time-locked manner^56^. The mechanisms underlying the neural encoding of speech were suggested to reflect both the enhancement of the attended stream and suppression of the unattended one^49^. Our recent study^50^ showed that the nature of the interfering stream significantly modulates attentional encoding, with fully-intelligible distractors causing the strongest encoding of both attended and unattended streams and latest dissociation between them, and non-intelligible distractors causing weaker encoding and earlier dissociation.

## Current Study

The current study used neural encoding of the speech envelope to investigate whether and how bilingualism modifies the mechanisms of auditory selective attention. Following our previous study^50^ we employed a cocktail-party paradigm, in which participants attended to a narrative in their native language presented to one ear, while ignoring a competing talker in the other ear. By manipulating the type of competing streams, we created interference at different levels of intelligibility. In the first condition, the interfering narrative presented in the unattended ear was also in the participant’s native language (Native-Native condition), arguably creating the most distracting listening environment. In the second condition, the interfering narrative was also linguistic in nature but in a language that participants did not understand (Native-Unknown condition). In the third condition the interfering stream was Musical Rain (MuR), a non-linguistic stimulus closely matched to the acoustic properties of speech that does not trigger speech percept (Native-MuR condition). Finally, the fourth condition was the ‘Single Talker’ condition, where participants attended to a narrative presented to one ear, with no interference presented to the other ear.

Based on the existing evidence^49,57,58^ we predicted that attention would increase speech encoding in all conditions compared to the non-attended stream. Furthermore, following the results from monolingual listeners presented with the same types of interference^50^, we hypothesized that the nature of the interfering stream might further modify attentional encoding, with intelligible interference (which is most difficult to dissociate from the attended stream) triggering late dissociation and strong enhancement of the attended stream. However, if the demands of learning and using multiple languages from an early age can indeed modify the mechanisms of selective attention, we could also expect a different pattern of results to that seen in monolinguals. This might be manifested in different timing of dissociation between attended and unattended streams, or different distribution of attentional capacity needed to achieve this across conditions – both potentially reflecting reconfiguration of the underlying mechanisms of focusing on the attended stream and distinguishing it from interference. In line with evidence that the brain adapts to the environmental demands to enable task performance^35^ we assumed that any such changes to the neural mechanisms of selective attention in bilinguals would serve to enable their optimal behavioural performance in this arguably more challenging processing environment, rather than to provide a behavioural advantage to bilinguals over monolinguals. In order to make this inference however, and ensure that any differences between monolinguals and bilinguals are not driven by differences in behavioral performance (which may or may not exist^29,31^), it was necessary to keep the task demands such that both groups are able to perform optimally and equally well. We therefore simply asked the participants to listen attentively and then answer comprehension questions after the recording of neural activity has taken place.

## Effects of Language Similarity

Finally, the current study also explored whether the typological similarity between the bilingual’s two languages plays an additional role in modifying the mechanisms of selective attention. Typological similarity is similarity in structural and functional features between languages, describing their commonalities in the phonological, lexical or syntactic domain. Whilst there is no universally accepted index of language similarity, and the outcome of any comparison depends on the specific criterion used, it is widely acknowledged that languages within the same genus (e.g., English and Dutch, both belonging to the Germanic genus of the Indo-European family) are more similar than those from different language genera (i.e., Slavic, Romance, Germanic). We therefore adopted a widely accepted classification^59^, which uses the typological similarity in phonology, vocabulary and grammar to classify languages within families or genera. On this basis, we selected to compare bilinguals whose languages either belong to the same genus of the Indo-European family (English and Dutch, both members of the Germanic genus) or a different one (English and Spanish, belonging to the Germanic and Romance genera respectively). Besides typological criteria, everyday experience attests that the vocabulary, inflectional systems and sound patterns of Dutch and English (including stress and intonation) are much more similar than that of Spanish and English, allowing Dutch learners to easily perceive and produce oral English, and acquire near-native accents. Table 1 lists experimental conditions for both groups of bilinguals.

**Table 1:** Experimental Conditions.

	Condition	Spanish-English Bilinguals		Dutch-English Bilinguals	
		Attended	Unattended	Attended	Unattended
1	Native - Native	Spanish	Spanish	Dutch	Dutch
2	Native - Unknown	Spanish	Serbian	Dutch	Serbian
3	Native - Musical Rain	Spanish	Musical Rain	Dutch	Musical Rain
4	Single Talker	Spanish	No interference	Dutch	No interference

The existing literature on the effects of language similarity on bilinguals’ cognitive performance is mixed. Some studies have shown that any combination of languages or dialects, irrespective of their typological similarity, alters the performance on tasks of attentional control and inhibition of unwanted information. For instance, a meta-analysis^60^ reported that bilingualism had a reliable effect on attentional control across language pairs as diverse as Chinese-English and French-English, while another study^61^ reported that Chinese-English, French-English and Spanish-English bilingual children all performed better than the monolingual controls on a colour-shape switching task, while showing no differences between the three groups. The same pattern was shown to hold even in cases of bidialectalism^62^, with speakers of two closely related varieties of Greek (Cypriot Greek and Standard Modern Greek) also performing better than monolinguals on tasks requiring switching and ignoring irrelevant information. However, a more recent meta-analysis^31^ found no evidence for the effects of bilingualism in general, and language similarity in particular, on the behavioral performance of bilinguals. Yet, whether and how language similarity might influence the neuroplastic changes to the mechanisms of selective attention in bilinguals remains unclear.

One hypothesis arising from the existing data is that, given the well-established parallel activation and competition between the bilinguals’ languages^14,27^, any combination of languages or dialects will modulate the systems that monitor for the presence of conflict and its resolution^29^. However, there is also evidence that competition between activated words can be modulated by variables like the degree of orthographic or phonological similarity between them, or the specific task that participants are performing^63–65^. For instance, while bilinguals generally recognize cognate words (i.e., words that share meaning and form across languages) faster than language-specific words, phonological overlap between words produces inhibitory effects in lexical decision tasks^63^, while cross-language orthographic similarity produces inhibitory effects when the task is to decide which language words belong to^64^. The alternative hypothesis is therefore that the degree of overlap between co-activated lexical entries can modulate the mechanisms of selection between them, triggering different activation patterns for selection between more similar ones (English and Dutch), compared to the more distant ones (English and Spanish). In this latter case, language similarity would emerge as another variable that helps fine-tune the underlying neural processes to enable optimal performance, without necessarily causing any apparent behavioural differences between the groups.

In sum, the current study investigated how the cognitive demands of using two languages modulate the neural mechanisms of selective attention, and whether the similarity between the languages plays a further role in shaping these processes. To this end, we tested how early Dutch-English and Spanish-English bilinguals encode attended speech in the presence of different types of interference, before comparing these results with the patterns observed in monolinguals using multivariate Representational Similarity Analysis^66^.

## Results

### Behaviour

Participants completed the comprehension task with a mean accuracy of 93.5% (SD = 4.9%) in the Spanish-English group and 88.68% (SD = 6.1%) in the Dutch-English group, indicating that the target speaker was attended to as instructed. One-way repeated measures ANOVA showed no difference between the number of correct responses across the four conditions in the Spanish-English group [F(3,63) = 1.38, *p* = 0.26], but significant difference between conditions in the Dutch-English group [F(3,51) = 6.46, p = 0.001]. Post-hoc t-tests showed that this was driven by the Single Talker condition, where the number of correct responses was lower than in the Native-Native and Native-MuR conditions (p < 0.05). This also affected the comparison of the overall performance in the two groups (t = −3.0, p < 0.01). Subsequent analyses however revealed that this unexpected Single Talker result in the Dutch-English group arose due to two ambiguous questions, where the majority of participants responded incorrectly. We also compared bilinguals with monolingual results we reported earlier^50^ (M = 94.3%, SD = 3.8%). Independent samples t-test showed that monolinguals and Spanish-English bilinguals did not differ from each other (t = 0.61, p = 0.54). However, monolinguals scored higher than the Dutch-English bilinguals (t = −3.9, p < 0.001).

### Effects of attention on neural encoding of speech

**Figure 1:**
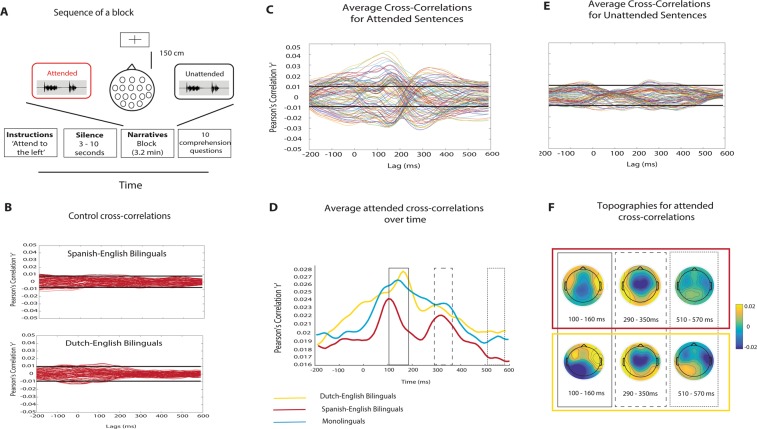
Experimental procedure and averaged cross-correlations. (**A**) Sequence of a block. Participants were instructed to attend to one side while fixating on a crosshair. The stimuli were presented 3–10 sec after the verbal instruction. After each block participants were asked to complete 10 true/false questions about the story they attended to. (**B**) Control cross-correlations between EEG channels and unrelated envelopes (Mel frequency cepstral coefficient) for each set. Black lines represent significance thresholds set at 97.5^th^ and 2.5^th^ percentiles. (**C**) Average cross-correlations for all attended sentences from −200 to +600msec post-onset. (**D**) Average of the absolute values of the attended cross-correlation function for the two bilingual groups (red and yellow) and monolinguals, blue. (**E**) Average cross-correlations for all unattended sentences from −200 to +600msec post-onset. (**F**) Topographies of the prominent latency ranges observed in (**D**) Warm colours = positive correlations, cool colours = negative correlations.

Across the two bilingual groups, continuous EEG data was recorded from participants listening to narratives in Spanish or Dutch, in four different listening conditions (Native Language, Unknown Language or MuR as interference, Single Talker). The first set of analyses aimed to establish the overall patterns of encoding to attended and unattended speech in bilinguals, and the extent to which this follows the pattern seen in monolinguals^50^. Cross-correlations for attended and unattended speech envelopes for bilinguals (averaged across participants and conditions) are depicted in Fig. 1. The attended cross-correlation functions (Fig. 1C,D) show robust neural encoding of the attended speech envelope, with major clustering of peaks around 100–150 ms and 300 ms post-onset, and a less prominent one around 550 ms; comparable to the results seen in monolinguals (overlaid in blue in Fig. 1D). The averaged cross-correlation functions for unattended speech (Fig. 1E) show that a limited number of EEG channels cross the significance threshold, indicating that attention had a major effect on encoding the speech envelopes in both groups. The shape of the unattended cross-correlation functions differs from the attended ones, replicating previous results^49,50^, and suggesting that the unattended cross-correlations are not a weakened representation of the attended ones. Scalp topographies for average attended cross-correlations (Fig. 1F) are plotted for latency ranges of 100–160 ms, 290–350 ms and 510–570 ms, based on the concentration of peaks at those time points. They are comparable across the two bilingual groups, with posterior central distribution of effects at earlier time windows, and more frontal distribution of the later effects.

### Comparisons across conditions: attended speech

One of the key findings in monolinguals^50^ was that the type of interference significantly modulated attentional encoding, with increasing intelligibility of the distractor causing stronger encoding of the attended stream (Native > Unknown > MuR); and Single Talker (no interference) condition triggering strongest attentional encoding overall. To assess whether the same pattern holds for bilinguals, we subjected attended cross-correlations (including the Single Talker condition) in each dataset to one-way repeated measures ANOVA, followed by pairwise post-hoc cluster-based permutation t-tests. In the Spanish-English group, the ANOVA results (FDR corrected for multiple comparisons) showed significant differences across conditions; post-hoc t-tests revealed that this was driven by the Single Talker condition, which showed strongest envelope encoding (Table 2). Importantly however, there were no significant differences between encoding of the attended streams across the three interference conditions (Native-Native, Native-Unknown, Native-MuR). In the Dutch-English dataset, a significant ANOVA followed by post-hoc t-tests again revealed that this was driven by the Single Talker condition, which differed from the Native-Native condition from 330 ms post onset. Once more however, post-hoc t-tests showed no significant differences between attentional encoding in the other three interference conditions.

**Table 2:** Cluster-based permutation t-tests between attended cross-correlation functions across conditions.

Attended vs Attended Comparisons										
*SPANISH - ENGLISH*	Positive Cluster					Negative Cluster				
	Onset (ms)	Peak (ms)	P value	T value	effect size	Onset (ms)	Peak (ms)	P value	T value	effect size
Native-Native vs Native-Unknown	n/a	n/a	N.S.	n/a	n/a	n/a	n/a	N.S.	n/a	n/a
Native-Native vs Native-MuR	n/a	n/a	N.S.	n/a	n/a	n/a	n/a	N.S.	n/a	n/a
Native-Unknown vs Native-MuR	n/a	n/a	N.S.	n/a	n/a	n/a	n/a	N.S.	n/a	n/a
Single Talker vs Native-Native	30	370	0.001	3340.8	0.4	190	320	0.001	−3512.2	0.4
Single Talker vs Native-Unknown	0	370	0.001	3980.3	0.5	20	450	0.001	−5009.7	0.5
Single Talker vs Native-MuR	n/a	n/a	N.S.	n/a	n/a	50	340	0.001	−3323.6	0.4
***DUTCH - ENGLISH***										
Native-Native vs Native-Unknown	n/a	n/a	N.S.	n/a	n/a	n/a	n/a	N.S.	n/a	n/a
Native-Native vs Native-MuR	n/a	n/a	N.S.	n/a	n/a	n/a	n/a	N.S.	n/a	n/a
Native-Unknown vs Native-MuR	n/a	n/a	N.S.	n/a	n/a	n/a	n/a	N.S.	n/a	n/a
Single Talker vs Native-Native	n/a	n/a	N.S.	n/a	n/a	330	560	0.016	−1329.7	0.9
Single Talker vs Native-Unknown	n/a	n/a	N.S.	n/a	n/a	n/a	n/a	N.S.	n/a	n/a
Single Talker vs Native-MuR	n/a	n/a	N.S.	n/a	n/a	n/a	n/a	N.S.	n/a	n/a

T value = sum of all t values within the cluster; Cohen’s d = effect size at cluster peak; n/a = absence of a significant cluster.

**Figure 2:**
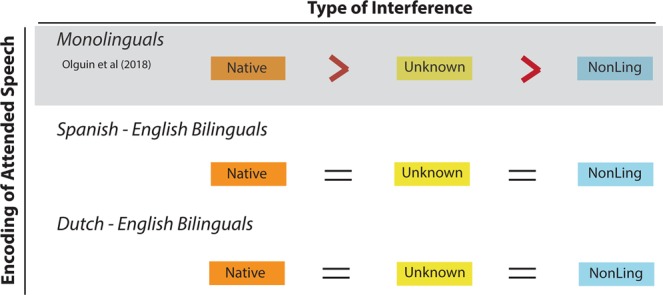
Summary of results for attentional encoding as a function of interference. Results reveal that monolinguals modulate the strength of attentional encoding as a function of the type of interference (native > unknown > non-linguistic), while neither group of bilinguals shows differentiation in the encoding of attended speech due to type of distractor.

This set of results conveys two key points: firstly, and consistently with the results in monolinguals, they show that selective attention requires processing capacity^12,67^ such that the presence of interference diminishes the capacity for entrainment to the attended stream, compared to the Single Talker (no interference) condition. More importantly however, they show that the nature of the distractor does not directly influence the strength of encoding of the attended stream in bilinguals. This is in stark contrast to the results from the equivalent analysis in monolinguals, which showed significant modulation of attentional encoding by the intelligibility of the interfering stream (Fig. 2). This clearly points to a modulation of selective attention mechanisms by the experience of speaking multiple languages.

### Comparisons across conditions: unattended speech

Next, we compared cross-correlation functions between the EEG data and unattended envelopes across the three interference conditions for both bilingual groups, following the same procedure as above. Results showed no significant differences between conditions in either of the groups, replicating the results seen in monolinguals, where only subsequent post-hoc analyses revealed subthreshold differences between unattended conditions. We explored such potential differences in the current data too, by comparing the unattended cross-correlation functions in each group using pairwise cluster-based permutation t-tests. In the Spanish-English group, the post-hoc t-tests showed no significant differences between unattended Native and Unknown streams, suggesting comparable encoding of unattended linguistic interference. However, both unattended linguistic interferences were more encoded than the unattended MuR stream (Table 3). In the Dutch-English bilinguals, all types of unattended interference were equally encoded, indicating no differences between encoding of unattended linguistic and non-linguistic interference.

**Table 3:** Cluster-based permutation t-tests between unattended cross-correlation functions across conditions.

Unattended vs Unattended Comparisons										
*SPANISH - ENGLISH*	Positive Cluster					Negative Cluster				
	Onset (ms)	Peak (ms)	P value	T value	effect size	Onset (ms)	Peak (ms)	P value	T value	effect size
Native-Native vs Native-Unknown	n/a	n/a	N.S.	n/a	n/a	n/a	n/a	N.S.	n/a	n/a
Native-Native vs Native-MuR	n/a	n/a	N.S.	n/a	n/a	0	60	0.005	−1804.29	0.9
Native-Unknown vs Native-MuR	10	280	0.002	2459.56	1.0	0	280	0.001	−3121.8	1.3
***DUTCH - ENGLISH***										
Native-Native vs Native-Unknown	n/a	n/a	N.S.	n/a	n/a	n/a	n/a	N.S.	n/a	n/a
Native-Native vs Native-MuR	n/a	n/a	N.S.	n/a	n/a	n/a	n/a	N.S.	n/a	n/a
Native-Unknown vs Native-MuR	n/a	n/a	N.S.	n/a	n/a	n/a	n/a	N.S.	n/a	n/a

T value = sum of all t values within the cluster; Cohen’s d = effect size at cluster peak; n/a = absence of a significant cluster.

### Comparisons within conditions: attended vs unattended speech

**Figure 3:**
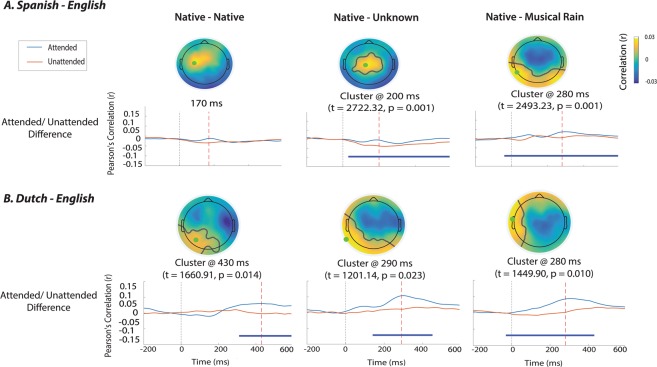
Comparisons of attended vs unattended cross-correlations in each condition. Results for (**A**) Spanish-English bilinguals and (**B**) Dutch-English bilinguals, showing topographies, timings and maxima of the clusters of significant difference. Horizontal blue lines denote the time window of significant differences between attended and unattended cross-correlations.

The next set of analyses aimed to establish the timing of dissociation of attended from unattended speech under different types of interference, by directly comparing attended and unattended cross-correlations in each condition separately. The equivalent analysis in monolinguals^50^ showed latest dissociation between the two streams when the interference was fully intelligible (the Native-Native condition), and differences right from the onset in the Native-MuR condition. In the Dutch-English bilinguals, these analyses showed a comparable overall pattern (Table 4), with the differentiation of attended and unattended streams emerging around 300 ms and peaking as late as 540 ms in the Native-Native condition; emerging around 150–200 ms and peaking at 300–400 ms in the Native-Unknown condition, and emerging right from the onset in the Native–MuR condition. Importantly however, the relative onsets of differentiation of linguistic interference in Dutch-English bilinguals are delayed by an average of 150 ms compared to the results seen in monolinguals. Spanish-English bilinguals also showed early differentiation of attended and unattended envelopes in the Native-MuR condition (starting from onset and peaking at 280 and 590 ms for positive and negative effects respectively), followed by the Native-Unknown condition (emerging at 30 ms and peaking at 200 ms). However, there were no statistically significant differences in this dataset between the encoding of attended and unattended streams in the Native-Native condition (Fig. 3 and Table 4).

**Table 4:** Cluster-based permutation t-tests between attended and unattended cross-correlations in each condition

Attended vs Unttended Comparisons										
*SPANISH - ENGLISH*	Positive Cluster					Negative Cluster				
	Onset (ms)	Peak (ms)	P value	T value	effect size	Onset (ms)	Peak (ms)	P value	T value	effect size
Native - Native	n/a	n/a	NS	n/a	n/a	n/a	n/a	NS	n/a	n/a
Native - Unknown	30	200	0.001	2722.3	0.8	n/a	n/a	NS	n/a	n/a
Native - MuR	0	280	0.001	2493.2	1.2	210	590	0.007	−1635.7	0.7
***DUTCH - ENGLISH***										
Native - Native	310	430	0.014	1660.9	0.8	270	540	0.011	−1751	1.8
Native - Unknown	140	290	0.023	1201.1	1	210	420	0.001	0.2262.5	1.2
Native - MuR	0	280	0.01	1449.9	0.8	0	590	0.001	−3902.7	1.4

T value = sum of all t values within the cluster; Cohen’s d = effect size at cluster peak; n/a = absence of a significant cluster.

These results reveal that, comparable to the results in monolinguals, the nature of interference affects how early the listeners can differentiate attended from unattended streams, with non-linguistic noise differentiated right from the onset, and linguistic interference differentiated later on. However, they also reveal that bilingualism, as well as the typological similarity of bilingual’s languages, modulate this process; with Dutch-English bilinguals showing evidence of delayed differentiation of the two types of linguistic interference, and Spanish-English speakers showing equivalent encoding of attended and unattended streams when the interference is in their native language.

### Attention over time

The continuous nature of stimuli allowed us to test whether effects of attention on neural encoding remain constant over time. To this end, we assessed the differences between the encoding of ‘beginning’, ‘middle’, and ‘end’ of each narrative across subjects. There were no significant differences in any condition between the strength of neural encoding over time (all p > 0.05) for either attended or unattended streams, indicating that the effects were constant throughout the narratives.

### Representational similarity analysis (RSA)

**Figure 4:**
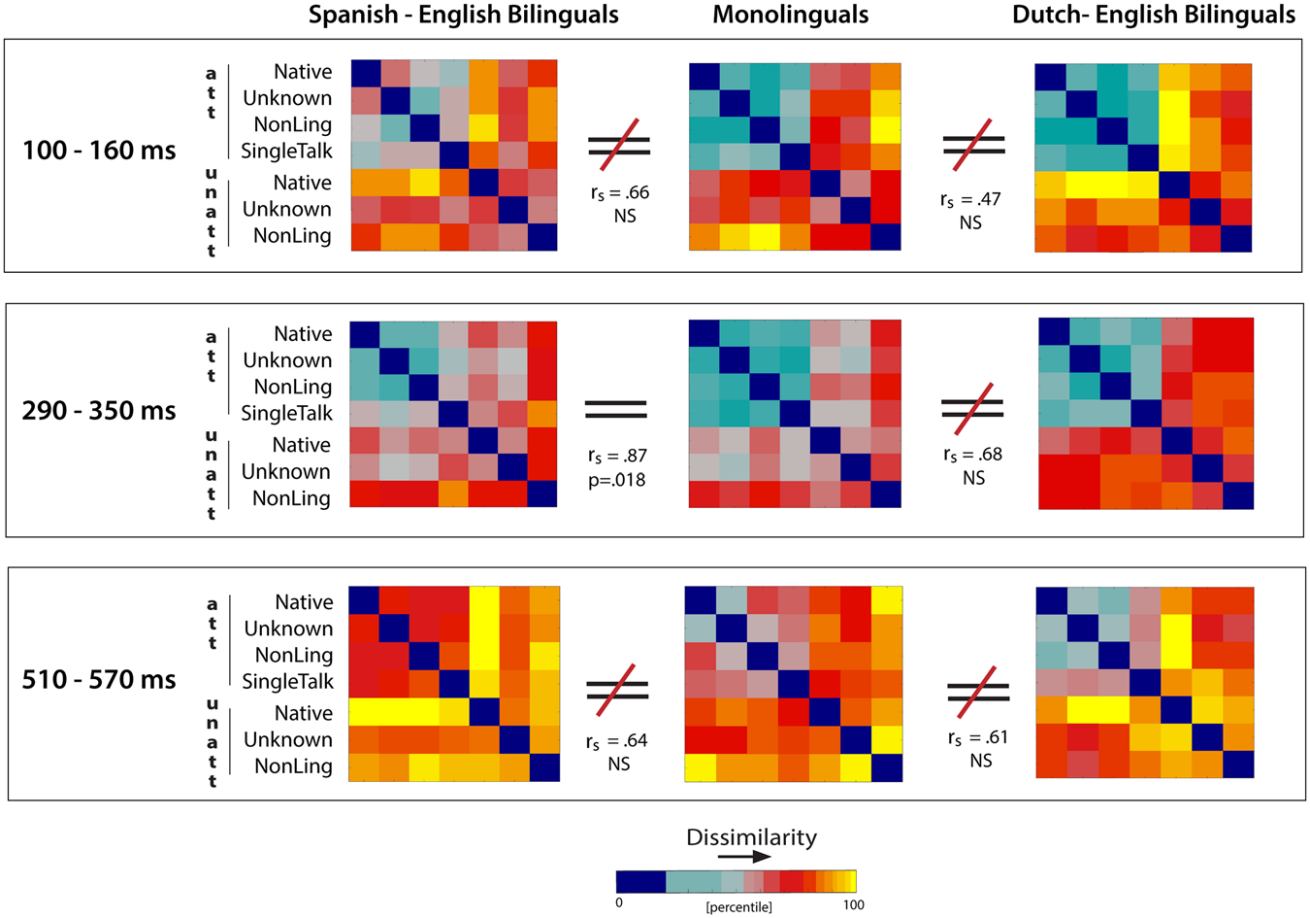
Multivariate RSA results. Direct comparisons of attentional encoding in bilinguals and monolinguals at three time windows of consistent effects (100–160, 290–350, 510–570 ms). Each RDM represents an experimental group at a particular time range.

The pattern of results reported above suggests that bilingualism modifies some of the key mechanisms of auditory selective attention, namely the strength of attentional encoding under different types of interference, as well as the timing of its differentiation from the unattended stream. To confirm these findings and directly compare attentional encoding across monolinguals and bilinguals - whilst superseding the unavoidable use of different stimuli in each group - we took advantage of RSA^66^, a multivariate pattern analysis that allows us to abstract away from the direct item-to-representation similarities and test for patterns of encoding in listeners presented with the same types of interference (second-order isomorphism). To this end, we extracted patterns of encoding for all attended and unattended conditions in each group, in the time windows of consistent attentional effects (100–160 ms, 290–350 ms and 510–570 ms, Fig. 1D). These patterns were compiled into 7 × 7 representational dissimilarity matrices (RDMs, one per time window per group) and compared within each window. The results are summarized in Fig. 4. As shown there, significant differences in the patterns of encoding emerged from the comparisons between monolinguals and bilinguals, with monolinguals differing from Dutch-English bilinguals at all time windows (100–160 ms, 290–350 ms, 510–570 ms) and from Spanish-English bilinguals in the early (100–160 ms) and late (510–570 ms) time windows. This adds support to the argument that bilingualism modifies mechanisms of selective attention, and that this modification to some degree reflects the typological similarity of the bilingual’s languages.

## Discussion

This study aimed to establish whether the demands of learning and using a second language influence the neural mechanisms of auditory selective attention, and whether this might be further affected by the typological similarity between the two languages. To this end, we tested the neural encoding of continuous attended speech in early balanced bilinguals of typologically similar Dutch and English, and typologically dissimilar Spanish and English, and compared them to results from English monolinguals reported earlier^50^. In a cocktail-party paradigm, participants attended to a narrative in their native language while ignoring a competing narrative in the other ear. The competing stream varied from fully intelligible story in the participant’s native language, to linguistic interference in a language unknown to the listener and well-matched non-linguistic noise (Musical Rain). The results clearly revealed that the experience of knowing and speaking multiple languages modulates the neural mechanisms of selective attention, even in the absence of consistent behavioural differences between monolinguals and bilinguals. They also suggested that the lifelong effects of the demands imposed by the typological similarly of bilinguals’ languages may help refine how the brain selects relevant information, tuning it towards the type of information recurrently used to dissociate between the co-activated languages. We elaborate on these findings below.

The neuro-cognitive consequences of bilingualism are a hotly-debated topic^32,68^. One controversial issue is how the experience of learning and using a second language affects the capacity to selectively attend to a stimulus in the presence of interference; with some studies reporting that bilinguals outperform monolinguals in such tasks^1,28,29^ and others questioning those findings^5,31,32^.Yet as argued earlier, it is unequivocal that learning and using multiple languages presents a major demand for our neurocognitive system, with parallel activation of both languages within the same network triggering competition and inhibition of the unwanted one^14,69^. Across domains as diverse as learning to juggle or read, memorising a sequence or acquiring detailed spatial knowledge, the brain responds to such environmental demands by neuroplastic adaptation and modulation of both its structural and functional architecture^33,34^. It is therefore unsurprising that similar effects have been observed in bilinguals too, with anatomical and structural changes including grey and white matter density^38,40,41^, connectivity^70^ or activation in the frontoparetial regions^43^, as well as altered processing of aspects of auditory information^6,44,46^. Our results complement these findings by showing that bilingualism modulates the neural mechanisms of selective attention, without necessarily causing any apparent behavioural differences between monolinguals and bilinguals.

The evidence emerged from both the analysis of how attended speech is encoded across different types of interference for each group separately, and from direct comparisons of activation patterns between monolinguals and bilinguals using multivariate RSA. In line with the literature^49,50,57,71^, the cross-correlation results showed that attention strongly modulated the neural tracking of speech envelopes, with stronger encoding observed for attended than for unattended speech. We also saw that Single Talker condition, where the attended stream was presented in the absence of any interference, triggered more robust encoding than attended speech in the interference conditions – replicating the findings that attention ‘consumes’ processing capacity^12,67^. However, and in stark contrast to the results observed in monolinguals^50^, the type of distractor did not have an effect on the strength of encoding of the attended stream in bilinguals. The finding that monolinguals enhance the tracking of the attended stream as interference becomes more intelligible^50^ conforms to the predictions of flexible accounts of selective attention^12,13^, where selection between streams will be less demanding when the distractor is non-intelligible and can be dissociated using lower-level perceptual information, while the dissociation between two fully intelligible streams requires the use of higher-level semantic and syntactic information, requiring more processing capacity and causing stronger encoding of the attended stream but delayed dissociation. However, this effect was not evident in either Spanish-English or Dutch-English bilinguals, both of which showed equal encoding of the attended streams across the three interference conditions. The RSA results further support these findings, with data showing that monolinguals differed from Dutch-English bilinguals in all time windows tested and from Spanish-English bilinguals in the early (100–160 ms) and late (510–570 ms) time windows, implying a modulation of both early and late attentional processing, where information is dissociated based on perceptual and lexicosemantic analysis respectively^12^. This complements the evidence that the type of interference - and the analysis it requires - does not impact attentional encoding in bilinguals the same way as it does in monolinguals. Yet despite the same overall pattern, some of the finer-grained results do not replicate across the two bilingual groups, suggesting that typological similarity of the bilinguals’ languages further shapes this neural modulation – a result to which we return later.

A possible reason for the lack of links between attentional encoding and the intelligibility of interference in bilinguals is that this reflects their ability to utilize fewer resources in difficult listening situations. This would be in line with the argument that consistent suppression of non-target language experienced by bilinguals leads to enhanced capacity for selective attention^26,72,73^. This practice might then reduce the attentional capacity needed for efficient encoding of the attended stream, which in turn would not vary as a function of the nature of interference – while still providing the basis for optimal behavioural performance across all interference conditions. Another possible explanation however links to the evidence that selective attention is a cognitive faculty with limited capacity. According to this interpretation, the process of selecting the target language and inhibiting the non-target one will unavoidably utilize some of the existing attentional capacity, thus limiting the resources available for further attentional enhancement as a function of the type of interference. As a result, there would be no increase in attentional encoding due to increase in intelligibility of interference – a pattern replicated in both Spanish-English and Dutch-English bilinguals. Either way, the present findings add to the substantial body of evidence about neuroplastic changes in response to environmental demands on our neurocognitive system, of which bilingualism is one prominent example. Yet, as previously noted^27^, one notable difference is that in many other domains the neuroplastic change is usually either closely related or in the same domain as the experience driving it (e.g., improved visuospatial coordination as a result of juggling^33^), while with bilingualism the effects go beyond language, extending into domain-general capacities like selective attention. Even more interestingly however, the current results show that this apparent modulation of neural mechanisms of selective attention in bilinguals does not necessarily result in changes to their behavioural performance. Put differently, our results suggest that bilinguals recruit mental resources differently from monolinguals in order to achieve the same performance, pointing to a different organization of the underlying neurocognitive mechanisms in the two groups.

The pattern of findings about the influence of language similarly on the way the brain selects relevant information is more complex. Here, the existing evidence is mixed, with some indicating that any combination of languages can modify bilingual’s performance on tasks requiring inhibition and attentional control^60,63^, consistent with the findings that both languages are activated in parallel regardless of language combinations, or even modalities (i.e., spoken and signed^74,75^); and other data contradicting these findings^32^. Since we were not interested in behavioural differences between the groups, and the task was designed to allow optimal and comparable performance across the board (i.e., simple comprehension), our focus was firmly on how varying demands of selection between more- or less-similar languages shape the underlying mechanisms of selective attention. In this context, language similarity is seen as an additional variable that helps fine-tune this neuroplastic adaptation. Our results suggest that there is indeed a subtle neural difference in the encoding of attended speech between bilinguals who speak a combination of typologically similar (Dutch-English) or dissimilar languages (Spanish-English). Despite the two groups being comparable in their absence of attentional boosting for intelligible interference, the Dutch-English bilinguals appear to show more comprehensive modulation of the underlying attentional mechanisms, with results showing differences across all three time-windows tested in RSA, and delayed dissociation of the two types of linguistic interference (where the comparable effects in monolinguals emerged 150 ms earlier on average^50^). This is particularly surprising for the unknown language interference (Serbian), as Dutch and Serbian belong to different genera of Indo-European family and have very different phonology, which should in principle be easy to differentiate for Dutch speakers.

This pattern is arguably pointing to the modification of the mechanisms of selective attention due to the life-long experience of interference from English to Dutch (where resolving competition might rely on stronger top-down processing), which we then see applied even when resolving interference from other languages. In other words, life-long experience with particular processing demands shapes attentional processing accordingly, such that Dutch-English bilinguals in this case use the strategy honed for dealing with their two similar languages, even with an interfering language that is less similar. This would be in line with the adaptive control hypothesis^76^, which suggests that control processes themselves can be adapted to the recurrent processing demands placed upon them. This modification is then just another example of adaptive changes of the mechanisms of selective attention by the demands of bilingualism – in this case the more specific variable of similarity between the co-activated entries. Whether this interpretation is correct or not, our findings suggest that the necessity to choose between typologically similar languages leads to more comprehensive modification of the mechanisms of selective attention, compared to the effects triggered by less similar languages. Another interesting difference between Dutch-English and Spanish-English bilinguals concerns the dissociation of attended and unattended speech in the Native-Native condition (Fig. 3). Here, Dutch-English bilinguals showed late dissociation of the interference in their native tongue as discussed above (starting from 270msec but peaking as late as 540 ms), while Spanish-English bilinguals encoded both attended and unattended native streams equally throughout the tested period. This surprising finding is most likely driven by strong encoding of unattended linguistic interference in the Spanish-English group (Table 3), which nevertheless did not impair their comprehension of attended narratives in this condition. Further research is however needed to clarify this.

In sum, this research revealed that bilingualism modulates the neural mechanisms of selective attention, with typological similarity of the two languages helping refine this process to reflect the requirements of resolving competition between more- or less-similar competitors. This is consistent with the view that learning and using multiple languages represents a major cognitive demand, which triggers neuroplastic adaptation of our processing system. The finding that this holds even in the absence of consistent behavioural differences between monolinguals and bilinguals shows that this reconfiguration is indeed adaptive in nature, aimed at allowing optimal behavioural performance. It also points to a different organization of the underlying neurocognitive mechanisms in early bilinguals, which may or may not be fully met or harnessed in the current educational systems – an intriguing hypothesis that requires further investigation. To our knowledge, this is the first study to investigate attentional encoding of natural continuous speech in bilingualism.

## Design and Methods

### Participants

Forty-six early bilinguals who learned English as their second language before the age of 6 were recruited from the University of Cambridge. Twenty-eight were native speakers of Spanish and 18 were native speakers of Dutch. Participants were recruited if they were balanced and fully proficient in both languages and did not report a dominant language. They completed the Bilingual Language Profile Questionnaire^77^, which assesses language dominance through self-report and takes into account age of acquisition, length of formal education in L1 and L2, environment where the languages are spoken, and dominance. There were no significant differences between the groups on any of these variables (p > 0.05; see Supplementary Materials for details). All participants were right-handed with no history of hearing problems. Six participants from the Spanish-English group were excluded from data analyses due to technical problems, thus 40 participants contributed to present study (17 males; mean age: 26.3). Participants were provided with detailed information regarding the purpose of the study and gave written consent. The study was approved by the Cambridge Psychology Research Ethics Committee and carried out in accordance with the relevant guidelines and regulations. The two groups of bilinguals were also compared to a group of 22 right-handed English monolingual listeners (10 males; mean age 21.5 years), whose results we reported earlier^50^.

### Stimuli and procedure

The stimuli for each group of bilingual listeners consisted of ten stories and two matched Musical Rain (MuR) sets that acted as a non-linguistic acoustic baseline. For the Spanish-English bilinguals, eight stories were in Spanish (native language) and two were in Serbian (language unknown to the participants, which belongs to the Slavic genus of Indo-European family). Two native Spanish female speakers recorded four stories each, and one native Serbian female speaker recorded the Serbian stories. Stories were simple children narratives, such as “Abdula y el genio”. For the Dutch-English bilinguals, eight stories were in Dutch (native language) and two were in Serbian (also unknown to the participants), recorded by female native speakers of the two languages. Gender was kept constant to reduce segregation strategies based on talker’s gender^78^. All stories were transcribed into 120 sentences each, with each sentence ranging from 2.5–3.1 seconds in length, and were normalised to have equivalent root mean square sound amplitude. From each story, the first 60 sentences (first half) were stringed together and the second 60 sentences (second half) were stringed together (with a 300 ms silence gap between each sentence), to create two blocks of approximately 3.2 minutes (192 s) in length. The full list of stimuli is presented in the Supplementary Materials.

The MuR acoustic baseline is a signal that closely tracks the acoustic properties of speech, while at the same time not being interpretable as speech^79^. To produce it we extracted temporal envelopes from the recorded stimuli and filled them with jittered fragments of synthesized speech. MuR thus preserves the spectrotemporal energy distribution, root mean square level, and the temporal envelope of the speech stimuli, but due to the absence of continuous formants it does not elicit speech percept. MuR was generated using MATLAB (The Mathworks Inc., 2010, Natick, MA, USA).

The study used a dichotic-listening task. In each condition, participants were instructed to attend to four blocks of stories (4 × 60 sentences, 240 sentences in total), which were counterbalanced between their left and right ear. A distractor stream was simultaneously presented in the other ear (Fig. 1A). Participants always attended to stories in their native language. There was no repetition of attended sentences (i.e., each sentence was attended to only once). The Single Talker condition was always presented first in order to familiarize the participants with the demands of attending left/right, and the remaining three conditions were presented in a random order. The order of stories within each condition was also randomized for each participant. In total, participants attended to 960 sentences across four conditions. The total number of unattended sentences was 720, due to the lack of interference in the Single Talker condition. This is the same experimental procedure as used in the study with monolinguals^50^, which we use for comparison with the bilingual data. For the duration of the experiment, participants sat in a comfortable chair in a sound-attenuated room. They were instructed to fix their gaze on a cross placed 150 cm in front of them. All stimuli were delivered through E-A-RTONE 3a earphones, with a mean intensity of 65 dB SPL, and presented using MATLAB’s Psychophysics Toolbox^80,81^. Prior to data acquisition we assessed the participants’ hearing using a short test which evaluated the perception of pure tones at different frequencies and dB levels. All participants achieved a 100% score on the hearing test.

### Behavioural measures

To ensure that participants were paying attention, keep the task requirements natural, and enable optimal behavioral performance, they were asked to simply listen attentively to the instructed side, and informed that they will be completing a set of comprehension questions after each block. There were ten yes/no questions after each block, for a total of 160 responses per participant.

### Data collection and preprocessing

We recorded EEG using 128 Ag/ag-CI channel electrode net (Electrical Geodesics Inc., Eugene, OR, USA). Thirty-six channels were excluded from the recording, as they are located in the outer layers of the net and measure significantly more muscle noise which is of no interest in the current study. Voltages for the remaining 92 channels were recorded at a sampling rate of 500 Hz, with net impedances kept below 100Ω. Data was down-sampled to 250 Hz, filtered between 1–100 Hz, and pre-processed in MATLAB: EEGLAB Toobox^82^. We epoched data at the sentence level (2 seconds) with a −200 pre-stimulus time window, which resulted in 960 attended and 720 unattended trials per participant. Artifact rejection was carried out per epoch, with bad trials removed and bad channels interpolated. In order to isolate independent components and identify artifacts such as eye blinks and non-brain activity, we used the Infomax Independent Component Analysis (ICA) algorithm. Artifacts were rejected according to their topography, time course, and spectral traits. Data was then re-referenced to the average of all channels.

### Speech envelopes

The temporal envelope of the speech was calculated for all attended and unattended stories and the MuR sets. Speech envelopes were computed using the Mel-frequency cepstral coefficients (MFCC). EEG data were down-sampled to 100 Hz to match the speech envelopes. The acoustic properties of the envelopes (i.e., the distribution of their mean frequency components) were matched across the three types of interference in both groups (F < 1; p > 0.05), ensuring validity of comparisons between them using the cross-correlation approach.

### Data analysis

The relationship between the EEG channels and the speech envelopes was characterized by calculating the Pearson’s correlation coefficient *r* as a function of lag. This procedure shows EEG activity that encodes the speech envelopes. If a speech envelope is in synchrony with an EEG channel at a particular latency, a non-zero cross-correlation will be shown at a lag equal to that latency. The cross-correlation function^83^ assumes a linear relationship between the acoustic envelope and neural activity, and has been widely used in the literature^49,84^. We calculated this correlation for each 10 ms lag in the range of −200ms before the onset of a sentence to 600 ms after the onset of a sentence, a time window that covers the range of the effects reported in the literature^85^. We cross-correlated the 92 EEG channels with the attended, unattended and control speech envelopes of each sentence. Control cross-correlations (which are due to chance, Fig. 1B) were obtained by cross-correlating speech envelopes of non-matching sentences with the EEG channels for each dataset separately. Control cross-correlation functions were then averaged across time and channels to form a Gaussian distribution, which was used to define the confidence interval at 95%. Attended and unattended cross-correlation values that were less than the 2.5^th^ percentile and more than the 97.5^th^ percentile were deemed to be significantly different from zero (p < 0.05, before correction for multiple comparisons).

In each dataset, we first computed average cross-correlation functions acorss all attended and all non-attended trials by averaging the correlation values for all participants and conditions at each time lag. This was followed by calculations of the attended and non-attended cross-correlation in each condition separately. The cross-correlation functions for all attended and all non-attended trials were not directly compared due to differences in the overall numbers of attended and unattended trials (960 vs 720). To test for differences between attended cross-correlation functions across the four conditions, we compared attended values per electrode in the −200 to 600 ms time window in a one-way repeated measures ANOVA, using a non-parametric permutation approach as implemented in the *statcond* function in the EEGLAB Toolbox. Control for multiple comparisons was achieved using False Discovery Rate (FDR p < 0.05)^86^ implemented in the *fdr_bh* function. The ANOVAs were followed by non-parametric cluster based permutation pairwise t-tests described below. The same approach was used to look at the differences between unattended cross-correlation functions across the conditions.

In order to evaluate the differences between pairs of attended or unattended cross-correlation functions, and also compare attended and unattended cross-correlation functions in each condition, we carried out non-parametric cluster-based permutation pairwise t-tests, as implemented in Fieldtrip MATLAB Toolbox^87^. To this end, pairs of experimental conditions were compared in 10 ms steps for each electrode in the −200 to 600 ms time window. All results with a t-value larger than 0.05 (two-tailed test) were clustered on the basis of temporal and spatial adjecency, and corrected for multiple comparisons using the Monte Carlo randomisation. Here, trials are randomly divided from a combined pool of two experimental conditions and placed into two subsets. To create a histogram of t-values and compute the proportion of random partitions with a value greater than the observed t-values, this process was repeated 1000 times. If the probability of the proportion (p-value) was less than 0.05, the conditions were considered to be significantly different from each other. For each cluster of significant differences we report T values (representing the summed t values across all significant electrodes) and effect size (Cohen’s *d*) at the peak. To calculate Cohen’s *d* we collapsed the relevant electrodes and time points (defined as 10 ms before and after the peak) into a vector of N participants for each dataset, and computed the difference between their means. This was done for each comparison in turn.

### Attention over time

To assess whether tracking of both attended and unattended acoustic envelopes changed as the story unfolded over time, we compared the neural encoding of sentences at the beginning, middle and end of the narrative. To this end, each block (60 sentences) was split into three equal parts consisting of 20 sentences (beginning: 1–20; middle: 21–40; end = 41–60), and then summed across all ‘beginning’, ‘middle’ and ‘end’ items per condition. This resulted in 80 sentences per group in each condition (e.g., condition 1 = 1a, 1b, 1c; where a = beginning, b = middle, c = end), which were compared for attended and unattended cross-correlations using non-parametric cluster-based permutation t-tests described above.

### Representational similarity analysis

To directly compare the patterns of neural encoding across the groups, we used Representational Similarity Analysis (RSA), a multivariate pattern analysis that examines the patterns of neural activity elicited by different experimental items^66^. At the heart of RSA is a distinction between first-order and second-order isomorphism^88^, where a first-order isomorphism captures resemblance between an item and its neural representation, while a second-order isomorphism captures the similarity structure of the items to the similarity structure of their representations. This allows us to abstract away from the direct item-to-representation similarities (which could be affected by different languages presented to each group) and look for similarities in the patterns of attentional encoding in bilinguals and monolinguals presented with the same types of interference. To this end, we used RSA to compute representational (dis)similarity matrices (RDMs) of cross-correlations observed for attended and unattended conditions in each group at time windows of consistent attentional effects (100–160 ms, 290–350 ms and 510–570 ms post sound onset, Fig. 1D). Each entry in an RDM represents dissimilarity (1 minus the correlation value) between activation patterns elicited by a pair of experimental conditions in a specific time-window, averaged across participants and electrodes. To determine the similarity of encoding patterns across the groups, we correlated the RDMs in each time window (Spearman’s ρ) and assessed these correlations against a null-hypothesis. The null hypothesis distribution of correlations was obtained by repeatedly randomizing the labels in one RDM and comparing it against the other. Correlations were deemed significant if they fell outside a 97.5% CI (one-tailed) after Bonferroni adjustment for multiple comparisons.

## Supplementary information

Supplementary Materials

## Data Availability

The datasets generated and analysed in the current study are available on request from the corresponding author.
