# Supplementary material for: Bilingualism and language similarity modify the neural mechanisms of selective attention
Source: Sci Rep. 2019 Jun 3;9:8204. doi: 10.1038/s41598-019-44782-3 (PMC6547874; doi:10.1038/s41598-019-44782-3)
Supplement: Supplementary file 1 — Supplementary Materials [file 41598_2019_44782_MOESM1_ESM.pdf]

Bilingualism and language similarity modify the neural mechanisms of selective attention

Andrea Olguin[*1], Mario Cekic[2], Tristan A. Bekinschtein[1], Napoleon Katsos[3], Mirjana Bozic[1]*

[1] Department of Psychology, University of Cambridge, Downing Street, Cambridge, CB2 3EB, UK

[2] Department of Computer Science and Technology, University of Cambridge, 15 JJ Thomson Ave, Cambridge CB3 0FD

[3] Department of Theoretical and Applied Linguistics, University of Cambridge; Sidgwick Avenue, Cambridge CB3 9DA

Supplementary Materials

1. Participant language profile

Participants were highly proficient bilinguals who reported learning L2 before the age of 6, and a fully balanced use of their two languages. For all participants, the second language was always English. Several participants reported that they also learned a third language in late childhood, but their knowledge was below an intermediate level in all cases. We also collected information about the length of their formal education in L2, L2 immersion and proficiency. The results are provided below, showing that the two groups were matched on all the variables tested.

	Spanish-English	Dutch-English	t-value	p-value
Formal English education in years	9.9	10.9	-0.59	.55
How long have they spent in an English speaking country	5.3	7.2	1.15	.25
English proficiency (self-rated on a scale 1-6)	5.7	5.8	1.27	.21
Years of experience with English	23.04	21.2	1.10	.28

The questionnaire did not explicitly measure how often the participants switch between the languages. However as all of them were Cambridge students and members of the University Spanish or Dutch societies respectively (which we used for recruitment), it is likely that the frequency of switching between their usage of English (for lectures, supervisions etc) and Spanish/Dutch (in a social context) would be highly comparable.

2. Stimuli for Spanish-English Bilinguals

Single Talker Condition

Hansel y Gretel

Block 1

1. Cerca de un bosque espeso y oscuro, **2.** Vivían un leñador y sus dos hijos. **3.** Los hijos se llamaban Hansel y Gretel. **4.** Su mamá había muerto hace muchos años. **5.** El padre volvió a casarse con una señora **6.** Ella no quería a Hansel y a Gretel. **7.** El padre cortaba leña en el bosque. **8.** Trabajaba con esfuerzo y sudor, **9.** Pero no ganaba el dinero suficiente **10.** para alimentar a toda su familia. **11.** Una noche, cuando Hansel y Gretel **12.** ya estaban acostados en sus camas, **13.** el leñador, triste, le dijo a su mujer **14.** "¿Cómo haremos para dar de comer a los niños? **15.** ¡No queda apenas nada de comida en la despensa!" **16.** "¡Pues, no les daremos de comer!" **17.** dijo la mujer muy ásperamente. **18.** "¡No tenemos dinero, son jóvenes y fuertes! **19.** Tendrán que buscarse la comida por su cuenta. **20.** Mañana los llevaremos al centro del bosque, **21.** ¡los abandonaremos allí y ya está!" **22.** El leñador alzó las manos horrorizado, **23.** "Abandonar a mis hijos en el bosque?! **24.** ¡Podrían ser devorados por los osos!" **25.** "Ya no tenemos con qué alimentarlos!" **26.** Dijo la madrastra con desprecio. **27.** El leñador intentó convencerla, **28.** pero cuando ella se enfadaba, **29.** él temblaba de miedo, **30.** así que al final accedió a su plan. **31.** Los niños se habían despertado **32.** al oír la discusión entre los adultos. **33.** Escucharon los crueles proyectos de la mujer, **34.** Para abandonarlos a los dos en el bosque. **35.** Gretel se puso a llorar, pero Hansel susurró, **36.** "No te preocupes, yo también tengo un plan".

37. Esa misma noche, cuando todos dormían, **38.** Hansel bajó silenciosamente por la escalera. **39.** Abrió la puerta y salió al jardín. **40.** Muchos guijarros blancos resplandecían **41.** a la luz de la luna en el césped. **42.** Se llenó rápidamente los bolsillos **43.** con bastantes guijarros blancos, **44.** y volvió a la cama muy silenciosamente. **45.** Por la mañana, su madrastra les dijo, **46.** "¿Qué les parece si fuéramos hoy al bosque?" **47.** Ustedes, podrían jugar, **48.** mientras yo ayudo a su papá a cortar **49.** leña para la casa, ¿no les gustaría?" **50.** Los niños no contestaron y echaron a andar. **51.** "Hansel, por qué vas tan despacio?!" **52.** gritó la madrastra mientras caminaban. **53.** Pero Hansel seguía retrasándose. **54.** Cada vez que la mujer le volvía la espalda, **55.** dejaba caer uno de los guijarros. **56.** Finalmente, después de dar muchas vueltas, **57.** Los padres se detuvieron entre los árboles. **58.** La madrastra les dijo fingiendo compasión, **59.** "Pobrecitos, deben estar cansados. **60.** Siéntense y descansen. Aquí tienen

Block 2
1. un poco de pan, para almorzar. **2.** Ahora esperen aquí hasta que regresemos." **3.** Los niños comieron el pan y jugaron. **4.** Se quedaron dormidos al pie de un árbol. **5.** Cuando despertaron, era ya casi de noche. **6.** "Nos han abandonado" sollozaba Gretel. **7.** "Ahora nunca volveremos a encontrar la casa!" **8.** Pero Hansel señaló la fila de guijarros. **9.** Tomó a Gretel de la mano y siguieron la pista **10.** de piedras blancas, hasta su casa. **11.** Cuando llamaron a la puerta, su papá abrió. **12.** Al verlos, los abrazó mil veces. **13.** Estaba encantado de que la cruel estrategia **14.** de su mujer no hubiera dado resultado. **15.** Pero la madrastra los miró con rabia. **16.** "Esto no les servirá de nada!" gritó ella. **17.** Mañana tenemos que perderlos en el bosque, **18.** y asegurarnos que no vuelvan a encontrar **19.** el camino de vuelta a casa. **20.** Los niños no se habían dormido aún, **21.** y oyeron las malévolas palabras **22.** y el cruel plan de su madrastra. **23.** Hansel se quedó tranquilamente acostado, **24.** hasta que todo el mundo estuvo dormido. **25.** Y entonces, bajó sin hacer ruido **26.** para recoger aún más guijarros. **27.** Pero esta vez la puerta estaba cerrada. **28.** Volvió a la cama y permaneció despierto **29.** Pensando cómo podría hacer esta vez. **30.** "¡Vamos!" gritó la madrastra por la mañana. **31.** "Pasaremos de nuevo el día en el bosque. **32.** Hansel, lleva el pan para el almuerzo." **33.** Hansel caminaba atrás de los demás. **34.** Cuando sus padres no le miraban, **35.** desmigajaba pedazos de pan por el suelo. **36.** "¡Avanza, Hansel!" gritaba su madrastra. **37.** "¡¿Por qué tardas tanto niño?!" le decía. **38.** Finalmente, llegaron al centro del bosque. **39.** Ahora, siéntense y coman su pan. **40.** Les había cansado tanto la larga caminata, **41.** que cayeron profundamente dormidos. **42.** Cuando despertaron, estaban solos. **43.** Pero esta vez, Gretel no lloró. **44.** "¿Dónde está la pista Hansel?" preguntó. **45.** "¿Cómo señalaste el camino esta vez?" **46.** "Con migas de pan!" contestó el hermano. **47.** "Pero ¿dónde están? No veo ninguna". **48.** No hallaron ni una sola de las migajas. **49.** Los pájaros se las habían comido todas. **50.** En el centro del oscuro y tenebroso bosque, **51.** Hansel y Gretel se abrazaron fuertemente. **52.** Los niños se arrimaron al pie de un árbol **53.** para protegerse del intenso frío. **54.** Los pájaros los miraban desde las ramas. **55.** "Nos hemos comido la pista de migas de pan, **56.** y ahora no pueden volver a su casa." **57.** Hansel y Gretel caminaron por el bosque. **58.** Con ojos atónitos, descubrieron una casa, **59.** construida con golosinas de varios colores. **60.** Los muros eran de un colorido mazapán.

Comprehension Questions

Block 1: *El padre no ganaba dinero suficiente para alimentar a toda la familia. (Y); El padre trabajaba como carpintero. (N); La mujer sugirió no darle de comer a los niños. (Y); La mujer planeaba abandonar a los niños en el bosque. (Y); El leñador estaba horrorizado con la idea de abandonar a los hijos. (Y); Cuando Hansel escuchó lo que la madrastra estaba tramando, se echó a llorar. (N); Hansel salió al jardín y se llenó los bolsillos de semillas. (N); Cuando caminaban por el bosque, se toparon con una serpiente. (N); Hansel dejaba caer los guijarros blancos cada vez que la madrastra les volvía la espalda. (Y); Después de dar muchas vueltas, la madrastra les dijo a niños que descansen. (Y)*

Block 2: *Hansel lloraba porque estaba preocupado. (N); Los niños siguieron la pista de guijarros blancos hasta su casa. (Y); El padre se enfureció al ver a los niños en la puerta de su casa. (N); La madrastra estaba feliz de ver a los niños de nuevo. (N); Hansel volvió a coger los guijarros blancos del jardín. (N); Hansel caminaba por delante de los demás. (N); Hansel decidió desmigajar el pan y dejar las migajas en el suelo. (Y); Los niños cayeron profundamente dormidos. (Y); Cuando despertaron, Gretel empezó a llorar. (N); Los pájaros se habían comido las migajas de pan en el suelo. (Y)*

El Oso y el Gnomo

Block 1
1. Erase una vez una pobre viuda **2.** que vivía en una pequeña casa en el bosque. **3.** En el jardín crecían dos árboles, **4.** uno de ellos tenía rosas blancas **5.** y el otro rosas rojas. **6.** Tenía dos hijas, y en honor a **7.** esos hermosos árboles, llamó a sus **8.** hijas Rosa Blanca y a la **9.** otra Rosa Roja y eran **10.** las niñas más dulces y buenas del mundo, **11.** siempre estaban alegres y ayudando **12.** a su madre en todo lo que les pedía. **13.** Rosa Blanca era mucho más tranquila **14.** que Rosa Roja que siempre estaba corriendo **15.** y jugando por los campos y prados. **16.** Le gustaba capturar mariposas. **17.** Rosa Blanca casi siempre se quedaba **18.** en casa con su madre y la ayudaba **19.** en el hogar, y le gustaba leer **20.** en voz alta algún cuento de hadas, **21.** cuando no había trabajo que hacer. **22.** Las dos niñas se querían mucho, **23.** y Rosa Blanca siempre decía: **24.** "Nosotras siempre estaremos juntas", **25.** a lo que contestaba,

Rosa Roja, **26.** "Estaremos juntas durante toda la vida", **27.** y la madre añadió: "Todo lo que una **28.** consiga deberá compartirlo con la otra." **29.** A menudo paseaban por el bosque **30.** recolectando bayas y frutas silvestres, **31.** y nunca, ningún animal **32.** les causó ningún daño. De hecho, **33.** los animales las amaban y confiaban **34.** en las dos chicas. La pequeña liebre **35.** comía una hoja de col de sus manos, **36.** el venado pastaba junto a ellas, **37.** el ciervo jugaba junto a ellas, **38.** y las aves se mantenían en las ramas **39.** y cantaban canciones para ellas. **40.** Nunca tenían ningún problema, **41.** si se entretenían y se les hacía de noche, **42.** no había problema, se quedaban **43.** a dormir sobre la hierba y dormían **44.** hasta la mañana siguiente, y **45.** su madre no se preocupaba, ya que **46.** sabía que todos los animales las querían **47.** y cuidaban; una mañana **48.** después de quedarse dormidas en el bosque, **49.** cuando se despertaron vieron a un **50.** hermoso niño con una túnica blanca **51.** resplandeciente, sentado cerca **52.** de su lugar de descanso. El niño **53.** se levantó, las miró amablemente, **54.** pero no dijo nada, y desapareció. **55.** Cuando miraron a su alrededor se **56.** dieron cuenta de que habían dormido **57.** cerca de un profundo barranco. **58.** Si hubieran ido un poco más allá **59.** durante la noche, habrían caído en él. **60.** Cuando le contaron a su madre la aventura

Block 2
1. ella les dijo que lo que habían visto **2.** era el ángel de la guarda de los niños. **3.** Rosa Blanca y Rosa Roja arreglaron la casa, **4.** y la dejaron limpia y ordenada. **5.** En verano Rosa Roja cuidaba la casa, **6.** y cada mañana, antes de que su madre. **7.** se despertara, ella colocaba un ramo **8.** de flores cerca de su cama, las rosas **9.** las cogía del árbol de rosas rojas **10.** de su jardín. En invierno, **11.** Rosa Blanca encendía el fuego **12.** y preparaba el té en la tetera de bronce, **13.** y también la frotaba y limpiaba **14.** hasta que brillaba como el oro. Por la noche, **15.** cuando empezó a nevar, su madre dijo, **16.** "Rosa Blanca cierra las ventanas," **17.** y se sentaron en torno al fuego, **18.** su madre se puso las gafas y empezó **19.** a leer un gran libro de cuentos de hadas, **20.** que eran los que más les gustaban **21.** a las dos chicas, que se dormían **22.** escuchando atentamente. Una noche **23.** cuando estaban sentadas junto al fuego, **24.** alguien llamó a su puerta, su madre **25.** dijo, Rosa Roja, por favor, **26.** mira quien llama a la puerta, debe ser **27.** algún viajero en busca de refugio. **28.** Rosa Roja fue corriendo hacia la puerta, **29.** pensando que habría un pobre hombre **30.** de pie, llamando a su puerta, **31.** pero no había un hombre, en la puerta **32.** había un oso, solamente un oso, **33.** y al Rosa Roja abrir la puerta, asomó **34.** su gruesa cabeza a través de la puerta. **35.** Rosa Roja gritó, y saltó hacia atrás **36.** aterrorizada, y Rosa Blanca **37.** se escondió detrás de la cama de su madre. **38.** Entonces el oso empezó a hablar, y dijo: **39.** "No tengáis miedo. Yo no voy a **40.** haceros daño. Estoy medio congelado **41.** y sólo deseo calentarme". **42.** "Mi pobre oso!", dijo la madre, **43.** "Ven, y acércate al fuego", **44.** Entonces ella llamó a sus hijas, **45.** y les dijo: "el oso no os va a hacer **46.** ningún daño – es una cariñosa criatura". **47.** Así que ambas salieron de sus escondites. **48.** Entonces el oso se tendió frente al fuego, **49.** y gruñó absolutamente feliz. **50.** Las niñas enseguida empezaron a jugar **51.** con él, y le hacían muchas bromas. **52.** El oso fue todas las noches a la misma hora, **53.** se acostaba junto a la chimenea **54.** y dejaba que las niñas jugaran con el. **55.** Cuando llegó la primavera, **56.** el oso les dijo, ahora me tengo que ir, **57.** "¿A dónde vas, querido oso?", **58.** "Tengo que ir a mi cueva y proteger **59.** mi tesoro de los gnomos malvadosn**60.** que salen al exterior para escavar y robar.

Comprehension Questions

Block 1: *En el jardín crecían dos arboles, uno con rosas blancas y el otro con rosas rojas. (Y); En honor a esos árboles llamó a sus hijas Rosa Blanca y Rosa Roja. (Y); Las dos niñas eran muy inquietas y no ayudaban a su madre. (N); Las dos niñas no se llevaban bien. (N); Las niñas paseaban por el bosque recogiendo flores. (N); Los animales amaban a las niñas y nunca les causaron daño. (Y); Si se hacía de noche, las niñas se quedaban a dormir en la hierba. (Y); Su madre siempre se preocupaba mucho. (N); Las niñas vieron a una hermosa mujer en túnica blanca. (N); La mujer en la túnica blanca les dijo que tengan cuidado con el barranco. (N)*

Block 2: *La madre leyó un libro de cuentos de hadas que eran los que mas les gustaban a las dos chicas. (Y); Una noche de invierno, alguien llamó a la puerta. (Y); La madre abrió la puerta. (N); Un venado estaba en la puerta. (N); Cuando Rosa Roja abrió la puerta, saltó hacia atrás, aterrorizada. (Y); El oso les dijo que no tengan miedo. (Y); El oso estaba congelado y deseaba calentarse. (Y); La madre estaba aterrada del oso y le dijo que se marchara. (N); Las niñas no querían jugar con el oso. (N); Cuando llegó la primavera, el oso dijo que se tenía que ir a su cueva a proteger su tesoro. (Y)*

Spanish-Spanish Condition

Abdula y el Genio

Block 1
1. Allí donde las arenas doradas del desierto **2.** lindan con el profundo mar azul, **3.** vivía una vez un pobre pescador **4.** llamado Abdula. Pasaba horas **5.** y horas en la playa echando **6.** su red al agua. La mayor parte **7.** de los días tenía suerte **8.** y pescaba algo. Pero **9.** un día la suerte le volvió la espalda. **10.** La primera vez que lanzó su red **11.** recogió un paquete de algas verdes **12.** y viscosas. La segunda, **13.** un montón de fuentes y platos rotos. **14.** Y la tercera, una masa **15.** de pegajoso limo negro. **16.** Abdula intentó sacar el tapón. **17.** Al fin, después de tirar de él **18.** durante un rato, lo consiguió **19.** y una bocanada de polvo se escapó **20.** de la botella. El polvo **21.** se convirtió

pronto en humo **22.** y tomó diversas coloraciones que **23.** empezaron a dibujar una forma: **24.** primero una cara, después **25.** un cuerpo… La figura creció **26.** y creció. En pocos segundos **27.** un enorme genio se elevó **28.** por encima del aterrado pescador. **29.** —¡Al fin libre! –rugió **30.** una voz más potente que el trueno **31.** -. ¡Libre después de tantos años! **32.** ¡Ahora, voy a devorarte! **33.** Abdula apretó la cabeza entre sus **34.** manos y gritó: -¿Por qué? ¡ **35.** ¿Qué os he hecho? ¡ Abdula lloraba. **36.** -¡Te cortaré en pedacitos! **37.** -exclamó el genio, al tiempo **38.** que mataba una bandada de pájaros **39.** que pasaba volando por encima **40.** de su hombro. -No lo hagáis, **41.** señor Genio - suplicó Abdula **42.** cayendo de rodillas - No quería **43.** molestaros. ¡Por favor, **44.** no me matéis! - Lloraba. **45.** -¡Te haré trocitos y te arrojaré **46.** a los peces! - vociferó el genio, **47.** que desenfundó una enorme espada **48.** con la que rozó la nariz del pescador. **49.** -¡Tened piedad! -lloró Abdula **50.** -¡Silencio! -tronó el genio. **51.** ¡Cállate y te diré por qué voy a matarte! **52.** Y sin retirar su espada del rostro **53.** de Abdula, el genio comenzó **54.** a contar su historia. **55.** "El gran Sultán Soleimán me encerró **56.** en esa botella para castigarme por **57.** los maleficios que realizaba en su reino. **58.** Me comprimió en esa horrible cárcel **59.** de vidrio y la arrojó al mar. **60.** He permanecido durante siglos

Block 2
1. en el oscuro fango. Lo único **2.** que oía era mi propia respiración. **3.** Lo único que sentía eran los latidos **4.** de mi corazón. Mi única esperanza **5.** era ser pescado y liberado **6.** por un pescador. Durante **7.** los mil años siguientes quedé **8.** quieto y pensé para mis adentros: **9.** Si logro salir alguna vez de esta horrible **10.** botella, mataré al primer hombre **11.** a quien vea. ¡Y después **12.** de él a todos los que me encuentre!" **13.** -¡Pero el Sultán Soleimán murió **14.** hace casi tres mil años! –gritó **15.** Abdula. -¡Exacto! -replicó **16.** con brusquedad el genio. **17.** - ¿Te sorprende que esté de tan pésimo humor? **18.** Profirió un gran grito, y el agua **19.** se puso a hervir en torno a sus tobillos. **20.** Levantó su gigantesca espada, que **21.** centelleó al sol, y cortó **22.** una nube en tiras encima de su cabeza. **23.** Luego miró hacia abajo para disfrutar **24.** por última vez del espectáculo **25.** del rostro aterrado del pescador. **26.** Pero Abdula no sólo no estaba asustado **27.** sino que permanecía de pie, **28.** con los brazos en jarras, **29.** la cabeza ladeada y la cara **30.** iluminada por una sonrisa. **31.** -Vámos, vámos, genio –dijo **32.** tranquilamente- Deja de tomarme **33.** el pelo y dime, de verdad, **34.** de dónde has salido. El suelo **35.** tembló cuando el genio inspiró profundamente. **36.** -¿Qué? ¡Tú, gusano! **37.** ¡Tú, inmundo bicharraco! **38.** ¡Prepárate a morir! Gritó. **39.** -¡Oh, vamos! Tú bromeas. **40.** Menudo cuento. Dime la verdad. **41.** Yo estaba distraído vaciando esa vieja **42.** botella y no te he visto acercarte. **43.** -¿Qué? ¡Yo he salido de esa botella! **44.** ¡Y voy a matar a todo el mundo! **45.** -Pero amigo mío, amigo mío **46.** suspiró Abdula- Tu madre **47.** nunca te enseñó a decir mentiras, **48.** sobre todo gordas. Basta ver **49.** el tamaño de esa botella y las dimensiones **50.** de tu cuerpo: tú has salido de esa **51.** botella tanto como yo. Entonces, **52.** Abdula, con grandes aspavientos **53.** hizo como que intentaba meter el pie **54.** por el estrecho cuello de la botella. **55.** -¡Tú, cucaracha! Tú… tú… **56.** El labio inferior del genio empezó a temblar. **57.** -¡Te digo que he salido de esa botella! **58.** -¡Puafl Ja Ja - se burló Abdula. **59.** ¡Entonces demuéstramelo! El genio, **60.** pensativo, decidió demostrárselo.

Comprehension Questions

Block 1: *El hombre se llamaba Jacobo. (N); Era un pescador. (Y); Cuando tiró del tapón, salió polvo y se convirtió en un genio. (Y); El genio creció y creció. (Y); El genio quería hacerse amigo del pescador. (N); El genio quería devorar a Abdula y cortarlo en pedacitos. (Y); El genio sacó una pistola. (N); Abdula no le temía al genio. (N); El genio sacó una enorme espada (Y); El rey Luis había encerrado al genio dentro de la botella. (N)*

Block 2: *El genio le contó a Abdula que había permanecido dentro de la botella por siglos. (Y); Su única esperanza era ser pescado y liberado por un pescador (Y); Se propuso que se volveria amigo del primer hombre a quien viese. (N); El Sultán Soleimán murió hace un año. (N); Cuando el genio levantó la espada, Abdula corrió a esconderse (N); Abdula tenía la cara iluminada por una sonrisa (Y); Abdula sí creía que el genio había salido de esa botella (N); Abdula le dijo al genio que su madre nunca le había enseñado a decir mentiras. (Y); El genio le había mentido a Abdula. (N); El genio se metió dentro de la botella para demostrarle a Abdula que sí había salido de esa botella. (Y)*

Barba Azul

Block 1
1. Érase una vez un hombre que tenía **2.** hermosas casas en la ciudad y en el campo, **3.** vajilla de oro y plata, **4.** muebles tapizados de brocado y carrozas **5.** completamente doradas; pero, **6.** por desgracia, aquel hombre tenía **7.** la barba azul: aquello le hacía tan feo **8.** y tan terrible, que no había mujer **9.** ni joven, que no huyera de él. **10.** Una distinguida dama, vecina suya, **11.** tenía dos hijas sumamente hermosas. **12.** Él le pidió una en matrimonio, **13.** y dejó a su elección que le diera la que quisiera **14.** Ninguna de las dos quería y se lo pasaban **15.** la una a la otra, pues no se sentían capaces **16.** de tomar por esposo a un hombre **17.** que tuviera la barba azul. **18.** Lo que tampoco les gustaba era que **19.** se había casado ya con varias mujeres **20.** y no se sabía qué había sido de ellas. **21.** Barba Azul, para irse conociendo, **22.** las llevó con su madre, con tres o cuatro **23.** de sus mejores amigas y con algunos jóvenes **24.** de la localidad a una de sus casas de campo, **25.** donde se quedaron ocho días enteros. **26.** Todo fueron paseos, partidas de caza **27.** y de pesca, bailes y festines, meriendas: **28.** nadie dormía y se pasaban toda la noche **29.** gastándose bromas unos a otros. **30.** En fin, todo resultó tan bien, que **31.** a la menor de las hermanas empezó **32.** a parecerle que el dueño de la casa **33.** ya no tenía la barba tan azul **34.** y que era un hombre muy honesto. **35.** En

cuanto regresaron a la ciudad **36.** se consumó el matrimonio. **37.** Al cabo de un mes Barba Azul dijo a su mujer **38.** que tenía que hacer un viaje a provincias, **39.** por lo menos de seis semanas, **40.** por un asunto importante. Le **41.** rogaba que se divirtiera mucho **42.** durante su ausencia, que invitara **43.** a sus amigas, que las llevara **44.** al campo si quería y que **45.** no dejase de comer bien. **46.** -Éstas son -le dijo- las llaves **47.** de los dos grandes guardamuebles; **48.** éstas, las de la vajilla de oro y plata **49.** que no se saca a diario; éstas, **50.** las de mis cajas fuertes, donde **51.** están el oro y la plata; ésta, **52.** la de los estuches donde están las pedrerías, **53.** y ésta, la llave maestra de todas las **54.** habitaciones de la casa. En cuanto **55.** a esta llavecita, es la del gabinete **56.** del fondo de la gran galería **57.** del piso de abajo: abrid todo, **58.** andad por donde queráis, pero **59.** os prohíbo entrar en ese pequeño gabinete, **60.** y os lo prohíbo de tal suerte que

Block 2
1. si llegáis a abrirlo, no habrá nada **2.** que no podáis esperar de mi cólera. **3.** Ella prometió observar estrictamente **4.** cuanto se le acababa de ordenar, y él, **5.** después de besarla, sube a **6.** su carroza y sale de viaje. **7.** Las vecinas y las amigas no esperaron **8.** que fuesen a buscarlas para ir a casa **9.** de la recién casada, de lo **10.** impacientes que estaban por ver todas **11.** las riquezas de su casa, pues no **12.** se habían atrevido a ir cuando estaba **13.** el marido, porque su barba azul **14.** les daba mucho miedo. **15.** Y ahí las tenemos recorriendo en seguida **16.** las habitaciones, los gabinetes, **17.** los guardarropas, todos a cuál **18.** más bellos y ricos. Después **19.** subieron a los guardamuebles, donde **20.** no dejaban de admirar la cantidad y la belleza **21.** de las tapicerías, de las camas, **22.** de los sofás, de los bargueños, **23.** de los veladores, de las mesas **24.** y de los espejos, donde se veía uno **25.** de cuerpo entero, y cuyos marcos, **26.** unos de cristal, otros de plata **27.** y otros de plata recamada en oro, **28.** eran los más hermosos y magníficos **29.** que se pudo ver jamás. No paraban **30.** de exagerar y envidiar la suerte **31.** de su amiga, que, sin embargo **32.** no se divertía a la vista de todas aquellas **33.** riquezas, debido a la impaciencia **34.** que sentía por ir a abrir el gabinete **35.** del piso de abajo. Se vio **36.** tan dominada por la curiosidad, que, **37.** sin considerar que era una descortesía **38.** dejarlas solas, bajó por una pequeña **39.** escalera secreta, y con tal precipitación, **40.** que creyó romperse la cabeza **41.** dos o tres veces. Al llegar **42.** a la puerta del gabinete, **43.** se detuvo un rato, pensando **44.** en la prohibición que su marido le había hecho, **45.** y considerando que podría sucederle **46.** alguna desgracia por ser desobediente; **47.** pero la tentación era tan fuerte, que **48.** no pudo resistirla: cogió **49.** la llavecita y, temblando, **50.** abrió la puerta del gabinete. **51.** Al principio no vio nada, porque **52.** las ventanas estaban cerradas; **53.** después de algunos momentos empezó a ver **54.** que en el suelo, habían restos **55.** de sangre de las mujeres anteriores **56.** que había tenido Barba Azul. Creyó **57.** que se moría de miedo, y la llave **58.** del gabinete que acababa de sacar **59.** de la cerradura, se le cayó de las manos. **60.** Recogió la llave y cerró la puerta.

Comprehension Questions

Block 1: *El hombre tenía barba color naranja. (N); El hombre era muy rico. (Y); Las mujeres huían del hombre porque tenia barba azul. (Y); La vecina tenía cuatro hijas hermosas. (N); El hombre quería casarse con alguna de las hijas. (Y); Las dos lo querían y se peleaban por el. (N); La mayor de las hermanas decidió casarse con él. (N); Barba Azul le dijo a su mujer que tenía que hacer un viaje corto de dos días. (N); Barba Azul dijo a su mujer que no podía invitar a nadie a casa (N); Barba Azul le prohibió a su mujer abrir el pequeño gabinete. (Y)*

Block 2: *Las amigas estaban impacientes por ver las riquezas de la casa (Y); Recorrieron toda la casa y admiraban todo (Y); La mujer sentía impaciencia por abrir el gabinete del piso de abajo. (Y); La mujer decidió no abrir el gabinete y quedarse con sus amigas. (N); La tentación de abrir el gabinete era tan fuerte, que no pudo resistirla. (Y); La mujer abrió la puerta del gabinete y al principio no vio nada porque las ventanas estaban cerradas. (Y); Barba Azul había acortado su viaje y se apareció en la casa. (N); La mujer comenzó a llorar descontroladamente pidiéndole perdón a Barba Azul por abrir el gabinete. (N); La mujer vio que dentro del gabinete habían muchos gatos. (N); La mujer dejó la llave caer al suelo por miedo, la recogió y cerro la puerta del gabinete. (Y)*

Spanish-Serbian Condition

El Agua Que Cura Todo

Block 1
1. Tres príncipes jóvenes veían cómo su padre, **2.** el rey, agonizaba en una cama gravemente **3.** enfermo. Ni siquiera los mejores **4.** curanderos de la región habían podido **5.** sanar al pobre rey, ninguna pócima, **6.** por mágica que fuera, **7.** le había devuelto la sonrisa. **8.** Un buen día mientras los tres muchachos **9.** caminaban entristecidos por el palacio, **10.** se apareció un anciano vestido **11.** con ropas andrajosas. Enseguida, **12.** dos de los príncipes quisieron echarlo, **13.** pero el menor de ellos se compadeció **14.** y le escuchó. – He sabido **15.** que vuestro padre ha enfermado terriblemente.**16.** Pero desde ahora les digo que lo único **17.** que podrá sanarle es el agua de la vida. **18.** Vayan a buscarla y lo podrán salvar. **19.** Al oír las palabras del anciano, los **20.** hermanos se llenaron de esperanza. **21.** El mayor de ellos partió rápidamente **22.** hacia su caballo y salió del castillo **23.** corriendo a toda velocidad. “Si obtengo **24.** el agua de la vida me ganaré el favor **25.** de mi padre para convertirme en rey”, **26.** pensaba el intrépido príncipe mientras **27.** se adentraba en el bosque. Justo **28.** en ese momento, se topó con un **29.** duendecillo que atravesaba el

camino. **30.** – ¿A dónde te diriges con tanta prisa, **31.** jovenzuelo? – preguntó la criatura. **32.** ¡No me molestes, estúpido! **33.** ¡Sal de mi camino! – gritó el príncipe **34.** sin detener su frenética marcha. **35.** Entonces, el duende se irritó tanto **36.** que lanzó un hechizo sobre el joven **37.** y lo hizo perderse entre las montañas. **38.** Con el paso del tiempo, el segundo **39.** de los hermanos comenzó a impacientarse. s**40.** "Si yo encuentro el agua de la vida **41.** mi padre me coronará como rey", murmuró **42.** el jovenzuelo mientras ensillaba su caballo **43.** y se desprendía galopando hacia el bosque. **44.** Nuevamente, el duende se cruzó **45.** en el camino del segundo hermano. **46.** – ¿A dónde vas con tanta prisa, jovenzuelo? **47.** – ¡Aparta, imbécil! – chilló el príncipe. **48.** – No tengo tiempo para tus preguntas. **49.** Y dicho aquello continuó su veloz carrera. **50.** El duende, molesto por la actitud del príncipe **51.** volvió a lanzar un hechizo para que se **52.** extraviara entre las montañas del bosque. **53.** Varias horas después, el más **54.** pequeño de los príncipes se preocupó **55.** por sus hermanos, pues aún **56.** no habían regresado con el agua de la vida **57.** para su enfermo padre. Sin pensarlo **58.** dos veces, ajustó su caballo y salió **59.** hacia el bosque. Por supuesto, **60.** el duende del bosque también vio al pequeño

Block 2

1. príncipe y decidió cruzarse en su camino. **2.** – ¿A dónde vas con tanta prisa, jovenzuelo? **3.** – Estoy buscando el agua de la vida **4.** para mi padre enfermo. ¿Sabes **5.** dónde puedo encontrarla? **6.** – ¡Claro que sí! – exclamó el duende **7.** con alegría al ver que, por fin, alguien **8.** le había tratado con amabilidad. **9.** –Debes buscarla en la cueva encantada. **10.** Pero ten mucho cuidado, porque **11.** un terrible oso protege la entrada. **12.** – Entonces, ¿cómo hago? – preguntó. **13.** – Toma este pan. Dáselo al oso **14.** y podrás entrar a la cueva. **15.** Antes que el oso termine de comer **16.** deberás haber salido. Date prisa. **17.** Y así lo hizo. El menor **18.** de los príncipes siguió el camino **19.** indicado por el duende y a las pocas **20.** horas arribó a la cueva encantada. **21.** Como le habían advertido, el oso **22.** se encontraba justo en la entrada. **23.** Era un animal enorme con garras **24.** afiladas y mirada furiosa, pero **25.** el príncipe hizo todo lo **26.** que el duende le había dicho. **27.** Cuando le lanzó el pan al oso, **28.** éste se entretuvo devorándolo y **29.** el príncipe se apresuró hacia el interior **30.** de la cueva. Todo se encontraba **31.** oscuro en aquel lugar, pero **32.** a lo lejos podía verse un manantial **33.** lleno de luz, y el joven no tardó **34.** en rellenar con aquella agua mágica **35.** un pequeño frasco que llevaba consigo. **36.** Justo antes de marcharse, el príncipe **37.** oyó una voz tierna que provenía desde **38.** lo lejos. Era la voz **39.** de una muchacha hermosa, con cabellos **40.** rubios que llegaban hasta el suelo. **41.** – ¿Quién eres? – preguntó **42.** el chico. – soy una princesa y **43.** he quedado atrapada en esta cueva. **44.** Por favor, sálvame. **45.** En ese momento, el príncipe recordó **46.** que no contaba con mucho tiempo, **47.** pues el oso estaba a punto de terminar **48.** con el pan. Besando las manos de **49.** la muchacha prometió regresar a buscarla, **50.** y se marchó de la cueva a toda carrera. **51.** Una vez en el bosque, el príncipe **52.** se encontró nuevamente con el duendecillo. **53.** – Amigo duende, debo agradecerte **54.** por todos tus consejos – dijo el príncipe **55.** – ahora mi padre podrá beber esta agua **56.** y curarse para siempre. – Me alegro **57.** que así sea, jovenzuelo – exclamó **58.** la criatura. – Ahora sólo me preocupan **59.** mis hermanos. Quisiera que volvieran **60.** a casa conmigo para celebrar.

Comprehension Questions

Block 1: *El rey estaba muy enfermo. (Y); Nadie lo había podido curar (Y); El rey tenía cuatro hijos. (N); Los hijos se toparon con una anciana vestida muy elegantemente. (N); El hijo menor se compadeció del anciano y lo escuchó. (Y); El anciano dijo que lo único que podría salvar a su padre era un té mágico. (N); El mayor de los hermanos fue el primero en salir del castillo para encontrar el agua de la vida. (Y); El hermano mayor se topó con una princesa en el camino. (N); El Segundo hermano se topó con un duende en el camino. (Y); El duende le dio el agua de la vida al segundo hermano. (N)*

Block 2: *Un águila se cruzó en el camino del príncipe menor. (N); El duende le dijo al príncipe menor dónde podía encontrar el agua de la vida. (Y); El duende le dijo que el agua de la vida se encontraba en una montaña. (N); Un terrible tigre protegía la entrada de la cueva. (N); El duende le dio pan al príncipe menor para que se lo de al oso. (Y); El príncipe hizo todo lo que el duende le había dicho. (Y); El príncipe rellenó un frasco con el agua mágica. (Y); El príncipe oyó una voz tierna. (Y); La voz le pertenecía a un unicornio blanco. (N); El príncipe le dijo a la princesa que no podría regresar a salvarla. (N)*

Bajka o Jasenki i Rabasku (Serbian)

Block 1

1. Teče reka Rabas između brda, **2.** šuma, livada i njiva. Žubori, šumi, **3.** negde utihne, na nekom mestu kao da se **4.** njegova voda svađa sa kamenjem koje je **5.** prekrilo dno. Sunce se probija kroz **6.** lišće drveća i, evo - već nekoliko **7.** najupornijih zraka uronilo je u rečicu, **8.** pozlatilo ribice i račiće i prozirna krila **9.** vilinog konjica koji je leteo nad vodom. **10.** Videli ljudi da je dobro imati put pored reke **11.** pa ga i napravili. Prolaze putem kad idu **12.** u vodenicu, u susedno selo poslom ili na veselje **13.** a i da se nađu sa prijateljima u teškim trenucima. **14.** Putem ide i daždevnjak u svom najlepšem **15.** crno-žutom odelu, a i zmija vijuga **16.** žureći u kamenjar. Tu su i srne koje **17.** sa srndaćem prelaze put da bi otišle **18.** u drugu šumu. Zastanu na putu, **19.** osluškuju nešto,onjuše, zatrepću **20.** svojim krupnim očima, iz kojih **21.** kao da će svakog trenutka kanuti suze, **22.** i odlaze dalje. Šuma kraj puta sanja **23.** i smeši se u snu. Ptice se ljuljuškaju **24.** na grani. Šumske životinje se pritajile. **25.** Samo leptir velikih šarenih krila, **26.** nečujno lebdi iznad cvetova kao da **27.** traži nešto. Dunu vetrić. Šuma se **28.** trže iz sna,

životinjice se pokrenuše, **29.** leptir pronađe cvet koji je tražio **30.** i sklopi krila. Vetar nešto šapnu šumi, **31.** a ona zatrese lišćem. Ptice zacvrkutaše **32.** na granama. Ču se žamor. Od cveta **33.** do cveta, od lista do lista, od grane do grane: **34.** "Šumska vila rodila kćerku! Šumska vila **35.** rodila kćerku!" Orilo se hiljadu glasića šumom. **36.** Pevalo se, veselilo. Dolete slavuj **37.** sa svojom porodicom i kad ču za novost, **38.** upita: "Kako se zove?" Tad se setiše **39.** da maloj vili treba dati ime. **40.** Beli jasenov cvet šapnu grani **41.** "Neka se zove Jasenka, jer je **42.** ovde rođena." Šapat se pretvorio u žamor. **43.** Svima bi po volji: Jasenka je **44.** lepo ime. Teče reka Rabas. Ribe, **45.** i velike i male, plivaju, iskaču **46.** iz vode, igraju se, talasići govore nešto **47.** jedan drugom, a žalosna vrba spustila grane **48.** do same vode da čuje kakvu to tajnu **49.** talasići imaju. I čula je: "Rodio se **50.** vodeni duh! Rodio se vodeni duh!" **51.** Radoznala žalosna vrba zatrese **52.** svojom dugom zelenom kosom i upita: **53.** "A kako se zove beba?" Rabas **54.** ponosno zažubori: "Rabasko, draga vrbo, **55.** Rabasko, tako sam srećan!" Vrba **56.** ništa ne odgovori, već uzdahnu a jato ptica **57.** polete. Tako se i u šumi i u reci proslavljalo **58.** zbog dve bebe: Jasenke i Rabaska. **59.** Raste mala šumska vila Jasenka. **60.** Raste i raduje se svemu što vidi, čuje, oseti.

Block 2
1. Razigrana, raspevana, omiljena. **2.** Peva sa pticama i cvrčcima, plače **3.** Sa malom bubamarom koja se izgubila. **4.** Pomaže pauku da isplete mrežu. **5.** Pentra se uz stabljiku šumskog cveta **6.** da pozdravi pčelu koja iz polena siše **7.** slatki sok. Bezbrižnu, nosi je leptir **8.** kad joj se prohte da leti. Iz prikrajka **9.** prate je nečije oči mračne i stroge. **10.** To je šumski vilenjak. Smeška se zlurado. **11.** Ne ume drugačije. Ne zna da se raduje. **12.** Svi ga se plaše i izbegavaju ga. **13.** On to zna i drago mu je što je tako. **14.** Sam, neuznemiravan. Samo da nije **15.** onih brbljivih ptica koje su savile gnezdo **16.** u blizini njegove pećine. Cvrkuću o ljubavi. **17.** Pih: ljubav! Važna je samo moć. **18.** Tako razmišlja zli vilenjak i čeka **19.** da Jasenka poraste. Uzeće je za ženu. **20.** Da Jasenka ne bi pristala, o tome **21.** ne misli, jer ko bi smeo i da pomisli **22.** da se suprotstavi njemu,koji je najstrašnije **23.** biće u šumi. Trudiće se da ona, **24.** kad postane njegova žena, bude **25.** okrutna kao i on. I zla, bez osmeha. **26.** To će biti prava stvar. Ovakva kakva je **27.** nije ono što on želi. Video je kad je **28.** pomagala jednoj maloj ljubičici da **29.** otvori latice. Dobrota je najveća glupost. **30.** A on,prepun mržnje i zla izlečiće je **31.** od dobrote i plemenitosti. Sa takvim mislima, **32.** zli vilenjak se povuče u tamu svoje pećine. **33.** Rabasko je bio nestašan vodeni duh. **34.** Skakao je sa kamena na kamen, **35.** sa grane a granu, sa grane u vodu. **36.** Plašio je ribe i račiće, oponašao **37.** žubor vode kad bi Rabas presušio **38.** pa su ljudi prolazeći putem, u čudu **39.** vrteli glavom i krstili se. Topao dan. **40.** Ni daška vetra, a lišće na vrbama **41.** treperi. To Rabasko plete vrbinu **42.** zelenu kosu. Smenjivali se dani i noći. **43.** Smenjivala se godišnja doba. **44.** Iza duge zime dođe proleće. **45.** Jasenka je trčala šumom. Sneg **46.** se topio i prvo prolećno cveće **47.** je pozdravljalo malu šumsku vilu. **48.** Jasenka, razdragana doskakuta do ivice šume. **49.** Rabasko se baš ljuljuškao na vrbinom listu, **50.** kad ugleda Jasenku. Iznenađen i ushićen, **51.** on kliznu sa vrbinog lista u Rabas. **52.** Talasići se nasmejaše, a ribe se zagnjuriše u vodu. **53.** Rabasko još jednom pogleda, protrlja oči, **54.** ali Jasenka je još uvek bila na ivici šume. **55.** I njeno srce je brzo, brzo kucalo, **56.** a znala je i zašto: zaljubila se. Poželela je **57.** da poljubi svaki cvetić, svaku travčicu. **58.** "Ovo je žena mog života!" pomisli Rabasko. **59.** "Sve ću učiniti da bude moja zauvek!" **60.** Šuma je znala a znao je i Rabas da je sve uzalud.

El Elfo del Rosal

Block 1
1. En el centro de un jardín crecía **2.** un rosal cuajado de rosas **3.** y en una de ellas, la más hermosa **4.** de todas, habitaba un elfo **5.** tan pequeñín, que ningún ojo **6.** humano podía distinguirlo. **7.** Detrás de cada pétalo de la rosa **8.** tenía un dormitorio. Era tan **9.** bien educado y tan guapo **10.** como pueda serlo un niño, y **11.** tenía alas que le llegaban desde **12.** los hombros hasta los pies. ¡Oh, **13.** y qué aroma exhalaban sus habitaciones, **14.** y qué claras y hermosas eran las paredes! **15.** No eran otra cosa sino los pétalos **16.** de la flor, de color rosa pálido. **17.** Se pasaba el día gozando de la luz **18.** del sol, volando de flor en flor, **19.** bailando sobre las alas de la inquieta **20.** mariposa y midiendo los pasos **21.** que necesitaba dar para recorrer todos **22.** los caminos y senderos que hay **23.** en una sola hoja de tilo. **24.** Son lo que nosotros llamamos las nervaduras; **25.** para él eran caminos y sendas, **26.** ¡y no poco largos! Antes **27.** de haberlos recorrido todos, **28.** se había puesto el sol; claro **29.** que había empezado algo tarde. **30.** Se enfrió el ambiente, cayó **31.** el rocío, mientras soplaba el viento; **32.** lo mejor era retirarse a casa. **33.** El elfo echó a correr cuando pudo, **34.** pero la rosa se había cerrado **35.** y no pudo entrar, y ninguna **36.** otra quedaba abierta. El pobre **37.** elfo se asustó un poco. Nunca **38.** había salido de noche, siempre **39.** había permanecido en casita, **40.** dormitando tras los tibios pétalos. **41.** ¡Ay, su imprudencia le iba a **42.** costar la vida! Sabiendo **43.** que en el extremo opuesto del jardín **44.** había una glorieta, recubierta **45.** de bella madreselva cuyas flores **46.** parecían trompetillas pintadas. **47.** Decidió refugiarse en una de ellas **48.** y aguardar la mañana. **49.** Se trasladó volando a la glorieta. **50.** ¡Cuidado! Dentro había dos personas, **51.** un hombre joven y guapo y una **52.** hermosísima muchacha; sentados **53.** uno junto al otro. Deseaban **54.** no tener que separarse en toda la eternidad; **55.** se querían con toda el alma. **56.** - tenemos que separarnos -decía **57.** el joven-. Tu hermano nos odia; **58.** por eso me envía con una misión **59.** más allá de las montañas y los mares. **60.** ¡Adiós, mi dulce prometida!

Block 2
1. Se besaron, y la muchacha, llorando, **2.** le dio una rosa, después de **3.** haber estampado en ella, **4.** un beso tan intenso y sentido que **5.** la flor se abrió. El elfo aprovechó **6.** la ocasión para introducirse en ella, **7.** reclinando la cabeza en los suaves **8.** pétalos fragantes; desde allí **9.** pudo oír perfectamente los adioses **10.** de la pareja. Y se dio

cuenta **11.** de que la rosa era prendida en el **12.** pecho del joven. ¡cómo palpitaba **13.** el corazón debajo! Eran tan **14.** violentos sus latidos, que el elfo **15.** no pudo irse a dormir. Pero **16.** la rosa no permaneció mucho tiempo **17.** prendida en el pecho. **18.** El hombre la tomó en su mano y, **19.** mientras caminaba solitario **20.** por el bosque oscuro, la besaba **21.** con tanta frecuencia y fuerza, **22.** que por poco ahoga a nuestro elfo. **23.** Éste podía percibir a través de **24.** la hoja el ardor de los labios **25.** del joven; y la rosa, **26.** se había abierto como al calor **27.** del sol más cálido de mediodía. **28.** Se acercó entonces otro hombre, **29.** sombrío y colérico; era el **30.** perverso hermano de la doncella. **31.** Sacando un afilado cuchillo de grandes **32.** dimensiones, lo clavó en el pecho **33.** del enamorado mientras éste besaba **34.** la rosa. Luego, le cortó **35.** la cabeza y la enterró, junto **36.** con el cuerpo, en la tierra blanda **37.** del pie del Árbol tilo. **38.** - aquí te quedarás, olvidado! **39.** pensó aquel malvado; "no volverá **40.** jamás. Debía emprender un **41.** largo viaje a través de montes **42.** y océanos. Es fácil perder **43.** la vida en estas expediciones **44.** y ha muerto. No volverá, **45.** y mi hermana no se atreverá **46.** a preguntarme por él." Luego, **47.** con los pies, acumuló hojas **48.** secas sobre la tierra mullida, **49.** y se marchó a su casa, a través **50.** de la noche oscura. Pero **51.** no iba solo, como creía; **52.** lo acompañaba el minúsculo elfo, **53.** montado en una enrollada hoja seca **54.** que se había adherido al pelo **55.** del criminal mientras enterraba **56.** a su víctima. Llevaba el sombrero **57.** puesto, y el elfo estaba temblando **58.** de horror y de indignación por **59.** aquel abominable crimen. El **60.** malvado llegó a casa al amanecer.

Comprehension Questions

Block 1: *Un elfo habitaba dentro de un árbol. (N); El elfo tenía alas. (Y); Las paredes pétalos de la flor. (Y); La rosa se había cerrado tras la puesta de sol y el elfo no pudo volver a entrar. (Y); El elfo no estaba asustando ya que siempre salía de noche. (N); El elfo decidió refugiarse en la casa de una anciana. (N); En el jardín había tres personas sentadas. (N); El hombre y la mujer se querían mucho. (Y); La pareja se tenia que separar. (Y); El hermano de la mujer quería mucho a la pareja. (N)*

Block 2: *La muchacha le dio una rosa al joven. (Y); El elfo aprovechó que la rosa estaba abierta para introducirse en ella. (Y); El joven coloco la rosa detrás de su oreja. (N); El joven echó la rosa al suelo. (N); El hermano de la mujer clavó un cuchillo en el pecho del joven. (Y); El joven gritó por ayuda y la muchacha vino enseguida. (N); El hermano le cortó la cabeza al joven. (Y); El hermano decidió no enterrar al joven y lo dejó expuesto. (N); El elfo temblaba de horror y de indignación por lo que había visto. (Y); El elfo le dijo al hermano que había visto todo y que le contaría la muchacha. (N)*

Mače Moni i mišić Boni (Serbian)

Block 1

1. Jasna je išla iz škole.Ranac na leđima **2.** bio joj je težak. Išla je lagano i razmišljala **3.** kako to da danas, onako lak zadatak **4.** iz matematike nije uspela da reši. **5.** Ide Jasna, gleda u vrhove svojih patika **6.** i vidi nešto – malo i žuto mače. **7.** "Ne na ulicu!" kaže Jasna, a mače **8.** gleda u Jasnu i mjauče jedva čujno. **9.** "Ti si maco gladna" kaže Jasna i **10.** vadi iz ranca kesu sa polupojedenom užinom. **11.** Mače halapljivo pojede ponuđenu užinu **12.** i, evo, već prede zadovoljno. Te večeri **13.** Jasna je unela mače tako da roditelji **14.** nisu primetili. "Baš si ti umiljata maca" **15.** kaže Jasna, a mače trlja glavicu o Jasninu nogu. **16.** "Kako da te zovem? pita Jasna macu **17.** ali izbor imena je, naravno, njen zadatak. **18.** Posle dužeg razmišljanja, odluči da će se **19.** mače zvati Moni.Sad je pola posla **20.** bilo gotovo.Ujutro će reći mami kako je **21.** mače bilo u njihovom vrtu. Mama voli životinje **22.** ali nije raspoložena da ih gleda **23.** kako šetaju po kući. Valjda će nekako **24.** uspeti da odobrovolji majku. Kada je **25.** sledećeg jutra Jasna sišla na doručak, **26.** čula je kako mama iznervirano govori tati **27.** "Zamisli, Bane, imamo miša u kuhinji! **28.** Pa to je strašno! Otkud on?! I to neki mali, **29.** ovolicki (pokazuje mužu od palca do kažiprsta)". **30.** "A imamo i mače" nadovezuje se Jasna, **31.** jer misli da je ovo pravi trenutak **32.** "ovolicko" (i ona pokazuje koliko je mače **33.** približavajući obe šake jednu drugoj **34.** kao da će da aplaudira). Tata poče **35.** da se smeje. "Pa ovaj dan je **36.** dobro počeo: imamo dva gosta!" **37.** Mama gleda u Jasnu koja, preskačući **38.** po dve stepenice, ode u svoju sobu **39.** i donese Monija. "E, draga, sad ti idi **40.** u kuhinju i donesi svog maleckog miša!" **41.** nastavlja tata šalu. Mama ćuti, ne zna **42.** da li da nastavi da se nervira zbog miša ili **43.** što je mače provelo noć u Jasninoj sobi. **44.** Jasna uzme šolju za kafu bez drške, **45.** usu malo mleka iz svoje šolje **46.** i stavi ispred Monija. Najzad, mama upita: **47.** "Otkud ti mače?" Ali tata opet **48.** spasi situaciju rekavši: "Ženo, **49.** danas spremaš ručak za petoro." **50.** Mama ne reče ništa, ustade i poče **51.** da sklanja ostatke od doručka. **52.** Jasna je sijala od sreće: mama je **53.** neće grditi, jer je tata, mada u šali, **54.** dao dozvolu da njen kućni ljubimac **55.** ostane u kući. Mišić se pojavi u **56.** trpezariji ali ga niko ne primeti, **57.** osim Monija. "Uzmi malo!" reče **58.** tiho Moni. "Ja sam mišić Boni i **59.** ne mogu s tobom da pijem mleko **60.** jer ti možeš da me pojedeš."

Block 2

1. "Neću, časna reč. Da sam hteo **2.** da te pojedem, mogao sam to odmah **3.** da uradim, a ne da s tobom pričam, **4.** zar ne?" "Biće da je tako" reče **5.** bojažljivo Boni, lagano se približavajući **6.** šoljici s mlekom. "Ja sam mače Moni **7.** i miševi mi nisu na jelovniku. **8.** Čuo sam od bake da smo nekad **9.** jeli miševe ali to je davna prošlost." **10.** Boni priđe i poče da pije mleko. **11.** "Hvala ti drugar, baš si kul! **12.** Ti si iz ove kuće, zar ne?" **13.** "Ne" reče Moni "donela me Jasna sinoć, **14.** a ti?" "Ne znam šta da ti kažem. **15.** Sinoć smo moji roditelji, braća,sestre i ja **16.** večerali. Onda

odjednom tata viknu: Beži! **17.** I ja sam bežao, bežao, bežao…"**18.** "Stani!" reče Moni. "Stao sam" uzbuđeno **19.** reče Boni "i jutros ja kod nekog šporeta, **20.** a žena viče:'Ju, ju, miš !' Otkud ja u kući, **21.** otkud kod šporeta – pojma nemam! **22.** Čudo! Kažem ti. Kakva je to kuća, **23.** nigde rupe da se zavučem u nju! I eto, **24.** sad pijem mleko sa mačkom! To je san!" **25.** "Ma, smiri se, Boni, ne sanjaš! Pij mleko!" **26.** "Kaži šta ja da radim, gde da idem, **27.** ne znam!" "Ne brini, Boni, sve će to **28.** ispasti dobro. Eto, živećeš ovde sa mnom.." **29.** "Jel?! Baš si duhovit, Moni! " **30.** Tebe će devojčica da mazi, **31.** ti ćeš da predeš, a ja? Ko će mene da mazi, a? **32.** Gde si video i čuo da miševi predu i da **33.** se umiljavaju!? Ko još mazi miševe?" **34.** "Možda si u pravu, a - možda i nisi." **35.** reče zamišljeno Moni. Jasna uze Monija **36.** u naručje i ode u svoju sobu. **37.** Tata i mama ostadoše da popiju kafu. **38.** Boni htede da pođe za Monijem ali odustade. **39.** Ode u kuhinju, šćućuri se iza šporeta i zadrema. **40.** Jasna je u svojoj sobi mazila Monija, **41.** a zatim uze sveske i knjige da radi **42.** domaće zadatke iz matematike i da uči. **43.** Moniju je bilo dosadno. Reši da **44.** malo odspava. Ali nije mogao. **45.** Razmišljao je o Boniju i njegovim rečima. **46.** Jadni Boni. Njega u ovoj kući niko **47.** neće voleti. Eto, on, Moni, je Jasnin **48.** kućni ljubimac. Onda mu sinu: a zašto **49.** Boni ne bi mogao da bude njegov **50.** kućni ljubimac?! Mama je otišla kod **51.** frizera, tata kod prijatelja na partiju šaha, **52.** a Jasna u školu. Moni siđe u kuhinju **53.** i tiho pozva Bonija. Boni izađe **54.** pitajući se u sebi da Moni nije promenio **55.** jelovnik. "Boni, imam ideju: ti ćeš biti **56.** Moj kućni ljubimac! Nije loše, šta kažeš, a?! **57.** Šta ti je, Boni? " Boni je zateturao, **58.** zakolutao očima, ali se, najzad malo smiri. **59.** "Ma, ništa, u redu je. To, to ti misliš ozbiljno?! **60.** Vežeš mi lančić oko vrata i pokazuješ me prijateljima! "

Spanish-Musical Rain Condition

El Niño Que Quería Un Arco Iris

Block 1
1. Juanito volvía andando de la escuela **2.** por un verde y delicioso valle. **3.** Ahí paseaban las ovejas y las vacas. **4.** Juanito siempre iba silbando por el valle. **5.** Él sabía silbar muchísimas canciones. **6.** Se acordaba de todas las canciones que escuchaba, **7.** porque había nacido en un molino de viento, **8.** en el momento en que el viento cambiaba del sur al oeste. **9.** También podía ver cómo soplaba el viento, **10.** y esto es algo que muy poca gente puede observar. **11.** Un día, al caminar hacia su casa, **12.** Juanito oyó al viento del oeste que se quejaba **13.** "Ay de mí! Ay, soplar! ¡La he olvidado!" **14.** "Qué es lo que has olvidado viento?" **15.** Estaba amargo, azul y tembloroso. **16.** "He olvidado mi canción favorita!" **17.** gritó el viento mientras soplaba. **18.** Juanito silbó una melodía y preguntó al viento, **19.** "es ésta tu canción, querido viento?" **20.** El viento se quedó encantado y le dijo, **21.** "Sí! ¡Esa es! ¡Qué listo eres Juanito!" **22.** Y revoloteó a su alrededor, jugueteando. **23.** "Juanito, te haré un regalo!" dijo el viento. **24.** "Una llave de plata y un riso de oro." **25.** "estas cosas no me sirven" pensó él. **26.** De modo, que se apresuró a decir **27.** "Por favor, preferiría un arco iris para mi solo." **28.** Y es que, con frecuencia, en el cielo de aquel valle **29.** Salían preciosos arco iris, aunque para Juanito, **30.** Siempre desaparecían demasiado pronto. **31.** "Un arco iris para ti solo es difícil" dijo el viento. **32.** "Toma un cubo, y ve caminando por el campo **33.** hasta que llegues al salto del pavo real. **34.** Llena el cubo de gotas de agua, tardarás bastante. **35.** Pero cuando lo tengas lleno, encontrarás dentro **36.** algo que pueda darte un hermoso arco iris. **37.** Por suerte, el día siguiente era sábado. **38.** Juanito cogió su almuerzo y un cubo, **39.** y caminó por el campo hasta las cataratas **40.** llamadas Salto del Pavo Real, en dónde el agua, **41.** al saltar por las rocas, formaba unas gotitas **42.** que resplandecían con unos colores maravillosos. **43.** Juanito permaneció todo el día en las cataratas. **44.** Recogía con el cubo las gotas de agua. **45.** Por fin, ya cuando se iba a poner el sol, **46.** tuvo todo el cubo lleno justo hasta el borde. **47.** Entonces, vio dentro del cubo algo que se movía **48.** y relucía con los brillantes colores del arco iris. **49.** Era un pececillo. "Quién eres?" dijo Juanito. **50.** "Soy el genio de la catarata. Échame otra vez al agua, **51.** y te recompensaré con un regalo" dijo él. **52.** "Sí" dijo el niño, "te echaré al agua. **53.** "Pero por favor, puedes darme un arco iris **54.** que me quepa en el bolsillo?" "mmm". **55.** "Te daré un arco iris, pero no es fácil de guardar. **56.** Creo que ni siquiera conseguirás llevártelo a casa. **57.** Pero si quieres uno, aquí lo tienes." **58.** El genio saltó del cubo, y se sumergió. **59.** Entonces, salió de las gotas de agua un arco iris, **60.** que fue a posarse en el cubo de Juanito.

Block 2
1. "Qué maravilla!" exclamó Juanito. **2.** Tomó el arcoíris con las dos manos sosteniéndolo **3.** como una bufanda y se quedó admirado **4.** de sus maravillosos y brillantes colores. **5.** Lo enrolló con cuidado y se lo guardó en el bolsillo. **6.** Luego emprendió el camino de regreso hacia su casa. **7.** Al atravesar el bosque, oyó que alguien lloraba **8.** escondido en un rincón oscuro entre los árboles. **9.** Se acercó para averiguar qué era **10.** y vio a un tejón que había caído en una trampa. **11.** "Querido niño!" gimió el tejón. "Déjame salir! **12.** O vendrán los hombres y los perros, y me matarán". **13.** "Me gustaría ayudarte, pero para abrir esa trampa, **14.** necesitaría una llave" dijo el niño. **15.** "Con la punta de ese arco iris que veo en tu bolsillo **16.** podrás forzar la puerta", dijo el tejón. **17.** Cuando Juanito empujó la punta del arco iris, **18.** la trampa se abrió y el tejón pudo escapar. **19.** "Muchas gracias!" masculló y desapareció. **20.** Juanito enrolló de nuevo el arcoíris. **21.** Se lo guardó cuidadosamente en el bolsillo. **22.** Pero los afilados dientes de la trampa, **23.** habían rasgado un gran trozo del arco iris. **24.** En el lindero del bosque, había una casita **25.** en la que vivía la vieja señora Benita. **26.** La señora Benita tenía muy mal carácter. **27.** Si por casualidad caía una pelota en su jardín, **28.** la cocinaba en el horno, hasta convertirla en carbón. **29.** Y todo lo que comía era de color negro. **30.** Pan quemado de negro y aceitunas negras. **31.** Llamó a Juanito y le dijo, "Oye chico, **32.** ¿me das un pedacito de ese brillante arco iris **33.** que te asoma por el bolsillo? **34.** El médico me ha recomendado que coma un pastel **35.** de

arco iris para curar mi enfermedad". **36.** A Juanito no le apetecía nada darle un pedazo. **37.** Pero la mujer parecía muy enferma. **38.** De mala gana, entró a la cocina, y le cortó **39.** un gran pedazo de arco iris con un cuchillo. **40.** Preparó una pasta dura con harina y leche hervida. **41.** Añadió el trozo de arco iris y cocinó la mezcla. **42.** Dejó enfriar el pastel, lo cortó en pedazos, **43.** Y se los comió con mantequilla y azúcar. **44.** Juanito también probó un trozo de pastel. **45.** "Mmmm" el pastel estaba delicioso! **46.** "Es lo mejor que he comido en todo el año!" **47.** dijo doña Benita. "Estoy harta del pan negro. **48.** Noto que este pastel me está sentando muy bien." **49.** Tenía mejor aspecto, se le colorearon las mejillas, **50.** Y empezó casi a sonreír de la felicidad. **51.** Juanito por su parte, después de haber comido **52.** su pedazo de pastel creció tres centímetros. **53.** "Más vale que no sigas comiendo" dijo la señora. **54.** Juanito guardó en el bolsillo el pedazo de arco iris. **55.** Cerca del molino de viento dónde vivía, **56.** su hermana Marita le salió al encuentro. **57.** Tropezó con una piedra, cayó al suelo, **58.** Y se hizo una herida que sangraba en la pierna. **59.** Marita, que sólo tenia cuatro años, **60.** empezó a llorar. "Mi pierna me duele mucho!"

Comprehension Questions

Block 1: *Juanito sabía silbar muchas canciones. (Y); El viento se quejaba porque había olvidado su canción favorita. (Y); Una nube le dijo a Juanito que le haría un regalo. (N); Juanito pidió un arco iris. (Y); Las cataratas se llamaban "Salto de la Vaca Real." (N); Juanito vio dentro del cubo algo que se movía. (Y); Lo que se movía dentro del cubo resultó ser una serpiente. (N); El pececillo le dijo a Juanito que era el genio de la catarata. (Y); El pececillo le dijo a Juanito que, si le daba de comer, lo recompensaría con un regalo. (N); El genio le regaló a Juanito un arco iris. (Y)*

Block 2: *Juanito guardó el arco iris en su bolsillo. (Y); Cuando atravesó el bosque, oyó que alguien lloraba. (Y); Un oso había caído en una trampa. (N); El tejón le dijo a Juanito que con la punta del arco iris podría abrir la trampa. (Y); En el bosque había un castillo. (N); En la casita vivía el viejo señor Luis. (N); La señora Benita le pidió a Juanito que le de un pedacito de su arco iris. (Y); El medico le había recomendado a la señora Benita que tome zumo de arco iris para su enfermedad. (N); Juanito engordó 5 kilos después de comer su pedazo de pastel. (F); La hermana de Juanito se llamaba Mónica. (N)*

Biancabella y la serpiente

Block 1

1. En las épocas de los reyes, de los duques, **2.** y los condes, hubo un marqués **3.** que no tenía hijos. Un día cualquiera, **4.** su esposa yacía descansando en el jardín, **5.** cuando una serpiente, sibilina **6.** y reptando entre la hierba, se deslizó **7.** hacia ella. No se le dio más **8.** importancia a tal evento hasta que, **9.** poco después, se supo la noticia **10.** de que se había quedado embarazada.**11.** El parto causó conmoción entre las matronas, **12.** quienes aseveraban que la niña que nació **13.** portaba una serpiente alrededor de su cuello, **14.** la cual se escapó sin atacar a nadie. **15.** La hija de los marqueses fue bautizada **16.** como Biancabella y tuvo una infancia feliz.**17.** Así fue hasta que un día, cuando **18.** contaba diez años, la serpiente **19.** se le apareció en el jardín, y empezó **20.** a hablarle. La serpiente le confesó, **21.** que era su hermana, Samaritana, **22.** y que si la obedecía sería feliz. **23.** De lo contrario se tornaría un ser miserable. **24.** La culebra le ordenó también **25.** llevar dos pozales, uno pleno **26.** de leche y otro de agua de rosas. **27.** Biancabella se angustió, y, **28.** de vuelta a su palacio, su madre **29.** sintió la pesadumbre en ella, **30.** y le preguntó. Sin responder, **31.** Biancabella pidió los dos cubos y, **32.** cuando se los hubieron llenado, los llevó **33.** al jardín. La serpiente **34.** le instó a bañarse dentro de ellos. **35.** A pesar de lo hermosa que Biancabella **36.** ya era, el baño le hizo más bella. **37.** Y, cuando su cabello fue peinado, **38.** de él se desprendieron joyas; **39.** y cuando sus manos fueron lavadas, **40.** de ellas se derramaron flores. **41.** Estos acontecimientos hicieron de Biancabella **42.** una joven todavía más atractiva, **43.** y los pretendientes se multiplicaron. **44.** Tras unas consultas y acercamientos, **45.** el padre de Biancabella, marqués, **46.** accedió al matrimonio de su hija **47.** con Ferrandino, monarca de un reino **48.** no muy lejano. Tras la boda, **49.** Biancabella buscó y llamó a Samaritana, **50.** su serpiente hermana, pero ésta no apareció. **51.** La recién casada se apenó porque pensó **52.** que había desobedecido a Samaritana, **53.** y triste buscó cobijo en su esposo. **54.** Por otro lado, la madrastra de Ferrandino, **55.** quien siempre había conspirado para casarlo **56.** con una de sus horrendas hijas, **57.** montó en cólera tras el casamiento **58.** Pasó el tiempo y Ferrandino **59.** hubo de partir a la guerra. Con él **60.** lejos, la madrastra llevó adelante su plan.

Block 2

1. y ordenó a sus sirvientes secuestrar y acabar **2.** con la vida de Biancabella, portándole **3.** una prueba de su muerte. Los sirvientes **4.** la raptaron y, aunque no la asesinaron, **5.** le sacaron sus ojos y le sajaron **6.** las manos. De esta manera **7.** podrían engañar a la madrastra sin haber **8.** acabado con la vida de aquella joven. **9.** La madrastra, pensando que su treta **10.** había salido como imaginaba, **11.** siguió adelante con sus designios **12.** y extendió por el reino el falso rumor **13.** de que sus hijas habían fallecido. **14.** Dicho rumor iba acompañado también **15.** de otra falsedad: que Biancabella **16.** había perdido un hijo que esperaba **17.** y que una fiebre la estaba debilitando **18.** de forma severa y casi irreversible. **19.** Una vez la mentira corría de boca en boca, **20.** la madrastra colocó a una de sus hijas **21.** en la cama de Biancabella. Ferrandino, **22.** tan pronto retornó de la contienda, **23.** sólo pudo que angustiarse. **24.** Biancabella, sin ojos ni manos, volvió **25.** a pedir ayuda a Samaritana, otra vez **26.** sin respuesta. Tuvo suerte **27.** de toparse con un anciano bondadoso, **28.** quien la quiso llevar de vuelta a casa **29.** a pesar de las reprimendas de su esposa, **30.** la cual al ver el estado de Biancabella **31.** daba por sentado que era una criminal

32. que había sido apropiadamente castigada. **33.** Biancabella solicitó a una de las hijas **34.** de este vetusto matrimonio que le peinase **35.** la cabellera, hecho que la anciana reprobó **36.** porque, como bien decía, su hija **37.** no era ninguna sirviente. A pesar **38.** de ello, la chica peinó a Biancabella, **39.** y resplandecientes joyas brotaron de su pelo. **40.** las alhajas sacaron a la honrada familia **41.** de la pobreza, y entonces sí **42.** que depositaron su confianza en Biancabella. **43.** Tras pasar un tiempo en su casa, la joven **44.** volvió a solicitar que la llevasen donde **45.** la habían encontrado, y esta vez **46.** todos accedieron de buen gusto. **47.** Una vez allí, Biancabella se desgañitó **48.** clamando por Samaritana, quien no **49.** aparecía ni a la de una, ni a la de dos, **50.** ni a la de tres. Tal fue la desesperación **51.** de Biancabella que pensó en el suicidio **52.** como vía de escape, y así hubiese **53.** procedido de no ser porque Samaritana **54.** irrumpió para salvarla. Fue entonces **55.** cuando Biancabella hubo de rogar el perdón **56.** de su hermana, la cual le devolvió los ojos **57.** y las manos. La magia no acabó ahí, **58.** pues la misma Samaritana se transformó **59.** en una mujer. Las hermanas **60.** regresaron con el matrimonio de los ancianos.

Comprehension Questions

Block 1: *La hija del marqués nació con una serpiente alrededor de su cuello. (Y); Biancabella tuvo una infancia muy triste. (N); Cuando Biancabella cumplió 15 años, la serpiente se le apareció. (N); La serpiente le dijo que era su hermana (Y); La serpiente dijo que se llamaba Rosa. (N); La serpiente le dijo a Biancabella que si le obedecía sería feliz. (Y); Cuando el cabello de Biancabella fue peinado, de él se desprendieron joyas. (Y); Los pretendientes de Biancabella se multiplicaron. (Y); El padre de Biancabella accedió al matrimonio de su hija con José. (N); La madrastra de Ferrandino era una buena persona y quería mucho a Biancabella. (N)*

Block 2: *Las sirvientas de la madrastra secuestraron a Biancabella. (Y); Siguieron las órdenes de la madrastra y la mataron. (N); Las sirvientas le sacaron los ojos a Biancabella. (Y); La madrastra extendió por el reino el falso rumor de que sus hijas habían fallecido. (Y); La madrastra extendió el falso rumor que Biancabella había tenido dos hijos. (N); La madrastra colocó a una de sus hijas en la cama de Biancabella. (Y); Biancabella sin ojos ni manos le pidió ayuda a Samaritana y ésta vino a ayudarla. (N); Biancabella se topó con un duende. (N); Biancabella pensó en el suicidio como vía de escape. (Y); Samaritana no perdonó a Biancabella y la dejó sin ojos y sin manos. (N)*

3. Stimuli for Dutch-English Bilinguals

Single Talker Condition

De Magische Klok

Block 1

1. Heel lang geleden woonden er op **2.** een prachtig kasteel zes koningszonen. **3.** Ze waren zo ijdel, dat ze bij de machtige **4.** tovenaar een klok besteld hadden, met **5.** zes ridders te paard, zodat iedereen die **6.** de klok zag, aan hen zou denken. **7.** Bovendien mocht de klok pas gaan lopen **8.** en de bel gaan klinken, zodra de **9.** koningszonen op hun trompet bliezen. **10.** Ze hadden de tovenaar beloofd hem **11.** daar drie zakken goud voor te geven. **12.** Maar toen de klok klaar was en ze **13.** bij hem kwamen om de klok te halen, **14.** hadden ze zoveel geld uitgegeven **15.** aan mooie kleren en lekker eten, dat **16.** ze nog maar één zak goud over hadden, **17.** en hem dus niet konden betalen. **18.** De tovenaar werd vreselijk boos. **19.** "Als jullie niet genoeg geld hebben, **20.** dan hadden jullie ook niet zo'n dure **21.** klok mogen bestellen," zei hij. "Maak **22.** nu maar gauw dat jullie wegkomen. **23.** Ik verkoop die klok wel aan iemand anders." **24.** En toen zette hij hen buiten de deur. **25.** Nu had Slimme Toontje, het knechtje **26.** van de tovenaar, achter de deur staan **27.** luisteren en zodoende alles gehoord. **28.** Aan deuren luisteren is een lelijk iets, **29.** maar Slimme Toontje had nog meer **30.** slechte eigenschappen. Hij kon liegen **31.** als de beste en was nog oneerlijk ook. **32.** Hij was alleen maar bij de tovenaar in **33.** dienst gekomen om stiekem achter alle **34.** geheimen te komen, in de hoop daar **35.** veel geld mee te kunnen verdienen. **36.** En hij verzon dan ook een heel kwaadaardig **37.** plan. De volgende dag zei de tovenaar tegen **38.** Slimme Toon: "Toon, Toon, ik moet op reis. **39.** Ik ga proberen of ik niet iemand kan **40.** vinden, die de Magische Klok wil kopen. **41.** Hier is de sleutel van m'n kamer; pas **42.** goed op dat er niemand binnenkomt."**43.** "Hèhè, niet zolang ik er ben," zei Slimme **44.** Toon en hij wuifde de tovenaar na tot **45.** die achter een heuvel verdwenen was. **46.** Toen rende Slimme Toon de trappen **47.** af en liep op een drafje het donkere **48.** bos door, naar het kasteel, waar hij **49.** zich bij de koningszonen liet aandienen. **50.** "Wat kom jij hier doen?" vroeg de **51.** oudste koningszoon. "Ik ben de **52.** knecht van de tovenaar," zei Slimme Toon, **53.** "en ik kom jullie vertellen hoe jullie **54.** voor één zak goud de Magische Klok **55.** kunnen krijgen. Als jullie mij die zak **56.** goud geven, dan zal ik de deur van **57.** de kamer van de tovenaar opendoen. **58.** Dan kunnen jullie de klok, nou, **59.** gewoon meenemen." "Hoeraaa!" **60.** riepen de zes koningszonen, en toen

Block 2

1. ze hoorden dat de tovenaar op reis **2.** was, nam één van hen Slimme Toon **3.** voor zich op z'n paard, en reden met **4.** z'n allen in galop naar de toren van de **5.** tovenaar. De koningszonen pakten daar **6.** de gouden wijzers, de wijzerplaat, **7.** de zes ruitertjes en de bel op hun **8.** paarden en reden terug naar het kasteel. **9.** Slimme Toon begon hard te werken. **10.** Hij metselde de wijzerplaat in het **11.** torentje van het kasteelplein. **12.** Daarboven kwamen de

ruitertjes. **13.** En helemaal op het dak van de toren **14.** bevestigde hij de bel. De koningszonen **15.** keken hun ogen uit. Oh, wat een mooie **16.** klok was dat. En ze hadden veel plezier **17.** dat ze die goeie tovenaar zó lelijk bij **18.** de neus hadden genomen. Toen de **19.** klok klaar was, gingen de koningszonen **20.** hun trompetten halen. "Blaas maar," **21.** zei Slimme Toon, "dan zul je eens wat zien." **22.** Dat deden ze, en ja hoor: de wijzers van **23.** de klok begonnen te draaien, de **24.** ruitertjes begonnen te rijden en de bel... **25.** Mis. De bel begon niet te luiden. Hoe ze **26.** ook bliezen, de bel deed het niet. **27.** De koningszonen werden vreselijk boos **28.** op Slimme Toon. Want de bel, ja, **29.** de bel vonden ze het mooiste van **30.** de hele klok. Maar... Slimme Toon **31.** had het al gezien: "Hèhè, we zijn de **32.** klepel vergeten," zei hij, "die ligt **33.** zeker nog in de kamer van de tovenaar. **34.** Uh, ik zal 'm wel even halen." **35.** De tovenaar was intussen de hele **36.** dag op stap geweest om de klok **37.** te verkopen. Maar hij had niemand **38.** kunnen vinden die zomaar drie **39.** zakken goud kon betalen. En hij **40.** was zó moe geworden van al dat **41.** geloop, dat hij met z'n rug tegen **42.** een boom was gaan zitten om wat **43.** uit te rusten. Plotseling zag hij in **44.** de verte een klein kereltje aankomen. **45.** "Wat vreemd," zei de tovenaar tegen **46.** zichzelf. "Dat lijkt Slimme Toon wel. **47.** Ik dacht dat hij op de klok zou passen, **48.** en nou loopt hij hier, door het bos?" **49.** Hij verstopte zich achter de boom, **50.** en ja hoor, daar kwam Slimme Toon **51.** voorbij, met de klepel op z'n rug. **52.** De tovenaar begreep er niets van, **53.** maar hij dacht wel dat er iets niet **54.** pluis was. En toen hij zag dat Slimme **55.** Toon de weg naar het kasteel insloeg, **56.** besloot hij hem ongezien te volgen. **57.** De zes koningszonen stonden **58.** al vol ongeduld te wachten, **59.** en ze hielpen Slimme Toon **60.** om op het torentje te klimmen.

Comprehension Questions

Block 1: *De zes koningszonen woonden in een landhuis. (N); Als de koningszonen hun trompet bliezen mocht de klok gaan lopen. (Y); De tovenaar vond het niet erg dat de koningszonen nog maar 1 zak goud overhadden. (N); De naam van het knechtje van de tovenaar was Slimme Toontje. (Y); Slimme Toontje was heel eerlijk. (N); Slimme Toontje verzon een goed plan om de tovenaar te helpen. (N); De tovenaar zocht iemand die de magische klok wilde kopen. (Y); Slimme Toontje rende door een weiland om het kasteel te bereiken. (N); Slimme Toontje wilde 1 zak goud voor de klok. (Y); De koningszonen waren blij met dit plan. (Y)*

Block 2: *Slimme Toontje zat op een paard. (Y); De koningszonen namen een trompet mee. (N); Onder de wijzerplaat kwamen de ruitertjes. (N); De koningszonen hadden veel plezier dat ze de tovernaar zo lelijk bij de neus hadden genomen. (Y); De koningszonen bliezen op hun trompetten. (Y); De bel ging rinkelen toen ze op hun trompetten bliezen. (N); De bel deed het niet omdat er geen lintje aan zat. (N); De tovenaar vond het vreemd dat hij Slimme Toon zag. (Y); Slimme Toon droeg de bel op zijn rug. (N); De zes koningszonen stonden geduldig te wachten, en ze hielpen Slimme Toon om op het torentje te klimmen. (N)*

De Boze Geest van Hoge Duvel

Block 1

1. Er was eens een boer uit Nunspeet **2.** die in Wiesel hakhout kwam halen. **3.** Maar toen hij de wagen had volgeladen **4.** en huiswaarts wilde keren, kon zijn paard **5.** niet voort en merkte hij dat het dier ziek **6.** was geworden. Zo kwam het dat hij zijn **7.** paard in Wiesel liet en een veearts ging **8.** halen, na eerst aan een daar wonende boer **9.** verzocht te hebben de wagen met hout voor **10.** hem naar Nunspeet te rijden. Deze boer **11.** zond zijn knecht met zijn paard. De knecht **12.** spande een zwart paard voor de wagen en **13.** reed naar Nunspeet. In de nacht keerde **14.** de knecht op het losse paard weer huiswaarts. **15.** Een enkele ster stond te pinken tussen wat **16.** zacht zwevende tere nachtwolken. Het was **17.** stil overal. De dorpjes en buurten welke **18.** hij door reed, lagen al te slapen tegen de **19.** heuvelhellingen. Het ketsen van de hoeven **20.** sloeg de stilte stuk en in het bos schreeuwde **21.** de bosuil: "Krie-oe, 'krie-oe." Dat hoorde **22.** de boerenknecht wel, maar hij was een **23.** onverschillige jongen en hij schreeuwde terug, **24.** zodat de slapende echo's er van wakker **25.** schrokken. Diep in de nacht kwam hij **26.** eindelijk bij 'Hoge Duvel'. Hij herinnerde **27.** zich de verhalen die hij gehoord had over **28.** de boze geest Ossaert die daar woonde **29.** en een plaag was voor heel de omtrek, **30.** totdat de heilige monnik - die aan het **31.** Uddelermeer woonde - met een ijzeren **32.** kruis de kwelduivel teruggedrongen had **33.** op 'Hoge Duvel' en waar hij 99 jaar moest **34.** blijven. De boer bij wie de knecht diende **35.** had de boze Ossaert wel in de struiken horen **36.** snurken en ook wel een blauw licht op **37.** 'Hoge Duvel' gezien. En de buurman had **38.** er eens honend geroepen: "Griepke, griepke **39.** grauw a'j' me griepen wilt, griep me dan **40.** gauw." Toen was er een groot zwart **41.** monster met vreselijke zwaarte boven op **42.** hem gesprongen. Hij had de klauwen in **43.** zijn rug gevoeld en dacht te zullen sterven. **44.** Hij had gelopen met de moed der wanhoop **45.** totdat het monster ineens losliet, omdat **46.** het niet verder dan 'Hoge Duvel' kon komen. **47.** De knecht geloofde van al die verhalen **48.** 'helemaal geen zak' en had er om gelachen. **49.** Nu hij de berg opreed wilde hij toch wel **50.** eens zien, wat er van al die onzin waar **51.** was. Gebeurde er iets, dan was het nog **52.** niet erg. Als hij aan de andere kant de **53.** voet van de berg bereikt had, zou de **54.** kwelgeest hem toch moeten loslaten. **55.** En boven op de berg gekomen riep hij **56.** uit alle macht: "Griepke, griepke grauw **57.** a'j' me hebben wilt, griep me dan gauw." **58.** Er sloeg een vlam uit de weg omhoog **59.** en een dreunende klap volgde. Het paard **60.** steigerde hoog, zodat de jongen - als hij

Block 2

1. een minder goed ruiter was geweest – er **2.** af gevallen zou zijn. Tegelijkertijd zag de **3.** knecht achter zich een groot zwart gevaarte **4.** op hem afkomen, dat met vurige klauwen **5.** naar hem greep. Hij zette het paard in **6.** draf en met de oren in de nek stormde **7.** het met zijn berijder de berg af. De boze **8.** Ossaert bleef achter. Wel was de ruiter **9.** wat geschrokken; maar zich bij de eik **10.** aan de voet van de berg veilig achtend, **11.** nu het monster hem niet verder volgen **12.** kon, keerde hij zich op het paard om **13.** en lachte de kwelduivel honend uit. **14.** Daar stootte de geest een woedend **15.** gebrul uit dat ver in het rond weerklonk **16.** en uit het bos dat ter weerszijden van **17.** de weg gelegen is en 'De Roode Heggen' **18.** genoemd wordt, sprongen een aantal **19.** weerwolven met groen lichtende ogen te **20.** voorschijn. Nu was het lachen uit en de **21.** roekeloze knecht werd door een grote **22.** angst bevangen. Het schichtige paard **23.** stormde als een wervelwind over de weg. **24.** De kop vooruit gestrekt, de neusvleugels **25.** trillend van angst. De galop van zijn **26.** neerbeukende hoeven sloeg de kluiten **27.** en keien uit de grond. Het werd een **28.** duivelse rit op leven en dood. In **29.** duizelingwekkende vaart sleurde het **30.** paard zijn berijder langs takken en struiken **31.** en de man zette het tot nog grotere spoed **32.** aan, tegelijk doodsangsten uitstaande, dat **33.** de krachten van het vermoeide dier uitgeput **34.** zouden zijn vóór het einde van de weg. **35.** De weerwolven huilden als een dolle **36.** verschrikking vlak achter hen aan; maar **37.** het paard won eindelijk iets op de **38.** achtervervolgers, die een vreselijk gehuil **39.** aanhieven en de vervolging sneller **40.** en sneller voortzetten. Nu scheen het **41.** paard uitgeput te zullen neerstorten. **42.** Telkens struikelde het, maar schoot **43.** dan weer vooruit. In de verte zag de **44.** ruiter de veilige hoeve al, maar de **45.** weerwolven wonnen. Hij hoorde **46.** hun gehuil steeds dichterbij komen **47.** en voor het laatst zette hij zijn trouwe **48.** paard tot uiterste spoed aan. Het dier **49.** wankelde, struikelde weer; de ruiter **50.** hield het nog op. De takken striemden **51.** de jongen in het gelaat. In een razende **52.** vaart reden ze het erf op en met de **53.** laatste en uiterste krachtsinspanning, **54.** droeg het kloeke dier zijn berijder tot **55.** op de deel. Het was net op tijd. Toen **56.** de knecht de grote deeldeuren snel **57.** dichtsloeg en er de boom voorschoof, **58.** waren de spookwolven op geen twee **59.** vadem meer van hem af. Hij hoorde **60.** hen buiten janken. Het trillende paard

Comprehension Questions

Block 1: *Het verhaal gaat over een visser uit Nunspeet. (N); Het paard van de boer kon niet voort en was ziek geworden. (Y); De knecht van de boer reed naar Nunspeet en keerde in de nacht weer huiswaarts. (Y); De knecht herinnerde zich verhalen over de boze geest Ossaert die bij 'Hoge Duvel' woonde en een plaag was voor heel de omtrek. (Y); De heilige monnik - die aan het Uddelermeer woonde - had de kwelduivel teruggedrongen op 'Hoge Duvel' met een gouden kruis. (N); De boer had de boze Ossaert in de struiken horen snurken. (Y); Er was een groot rood monster met vreselijke zwaarte bovenop de buurman gesprongen. (N); De knecht geloofde van al die verhalen 'helemaal geen zak' en had erom gelachen. (Y); Nu de boer de kuil inreed wilde hij toch wel eens zien, wat er van al die onzin waar was. (N); Er sloeg een vlam uit de weg omhoog en een dreunende klap volgde. (Y)*

Block 2: *De knecht zag achter zich een groot zwaar gevaarte op hem afkomen. (Y); De knecht keerde zich op het paard om en lachte de kwelduivel vriendelijk toe. (N); Uit het bos sprongen een aantal vossen met groenlichtende ogen tevoorschijn. (N); Het schichtige paard stormde als een wervelwind over de weg. (Y); Heel langzaam sleurde het paard zijn berijder langs takken en struiken. (N); De wolven huilden als een dolle verschrikking vlak achter hen aan. (Y); Telkens struikelde het paard, maar dan schoot het weer vooruit. (Y); De boer hoorde het gehuil van de weerwolven steeds verder weg gaan. (N); Heel langzaam reden het paard en de ruiter het erf op. (N); Toen de boer de grote deeldeuren snel dichtsloeg en er een boom voorschoof, waren de spookwolven op geen drie vadem meer van hem af. (N)*

Dutch – Dutch Condition

De Prinses Die Niet Kon Lachen

Block 1

1. Er leefde eens een koning die **2.** onmetelijk rijk was. Bovendien was **3.** hij zo vrijgevig als een priester zou **4.** moeten zijn, zo dapper als een **5.** leeuw en zo eerlijk als goud. Toch was deze **6.** koning niet gelukkig. Hij tobde dag **7.** en nacht en klaagde: "Geen galeislaaf **8.** lijdt zoveel als ik. Mijn enige kind, **9.** een dochter die zo mooi is als het **10.** daglicht en vromer dan een heilige, **11.** is altijd treurig. Niemand heeft haar **12.** ooit aan het lachen kunnen maken. **13.** Daarom wordt ze 'de prinses **14.** met het treurige gezicht' genoemd. **15.** In mijn stallen staan zevenhonderd **16.** prachtige paarden, stuk voor **17.** stuk zo zwart als een schoorsteen, **18.** maar ik houd alleen van mijn **19.** grote witte paard. En dat beest **20.** is zo kwaadaardig, dat zelfs de **21.** beste smid ter wereld niet in **22.** staat is alle vier zijn hoeven goed **23.** te beslaan. Daarom wordt mijn **24.** grote witte paard 'IJzerbreker' **25.** genoemd. Zelfs een galeislaaf heeft **26.** het niet zo moeilijk als ik." **27.** Toen het de koning te veel werd, **28.** ontboodt hij de stadsomroeper **29.** naar zijn paleis. "Omroeper, **30.** hier heb je duizend goudstukken. **31.** Roer de trom en maak overal **32.** bekend dat de man die de prinses **33.** met het treurige gezicht aan **34.** het lachen kan maken en het grote **35.** paard IJzerbreker aan vier hoeven **36.** kan beslaan, mijn schoonzoon **37.** en erfgenaam zal worden." **38.** "Tot uw dienst, edele koning." **39.** De trom werd geroerd en een **40.** grote hoeveelheid kandidaten **41.** die hun geluk wilden beproeven, **42.** meldde zich aan, maar ze moesten **43.** onverrichter zake terugkeren. **44.** Nu woonde er in die tijd in Fumel **45.** een flinke jonge smid bij zijn moeder. **46.** Op een avond zei hij tijdens het **47.** eten: "Moeder, morgen ga ik

naar **48.** de stad waar de koning woont om **49.** de prinses met het treurige gezicht **50.** aan het lachen te maken. **51.** En ik zal het grote paard Ijzerbreker **52.** aan al zijn vier hoeven beslaan. **53.** Zo zal ik de schoonzoon en de erfgenaam **54.** van de koning worden." "Goed, mijn **55.** lieve zoon, ga naar de stad van **56.** de koning en moge God je leiden." **57.** Toen de moeder naar bed was, **58.** haalde de jonge smid uit een **59.** kist zijn hele vermogen te voorschijn: **60.** 100 zilverstukken en 50 goudstukken.

Block 2

1. Van de 100 zilverstukken smeedde **2.** hij 4 zilveren hoefijzers. Van de 50 **3.** goudstukken smeedde hij 28 gouden **4.** spijkers, 7 voor elk hoefijzer. Tegen **5.** de morgen was alles klaar. De smid **6.** stopte een brood, een fles wijn, **7.** een hamer, de vier zilveren hoefijzers **8.** en de achtentwintig gouden spijkers **9.** in zijn leren tas. Vervolgens vertrok **10.** hij met de tas over zijn schouder **11.** naar de stad van de koning. Toen **12.** hij drie uur had gelopen, ging hij **13.** aan de kant van de weg zitten **14.** om te eten en te drinken. In een **15.** nabijgelegen korenveld sjirpte een **16.** krekel die zo zwart was als roet uit **17.** een schoorsteen: "Kri, kri, kri, goedemorgen **18.** beste smid." "Goedemorgen mijn **19.** beste krekel, wat is er van je dienst?" **20.** "Kri, kri, kri, beste smid, vertel me eens, **21.** waar gaat de reis naartoe?" "Mijn **22.** beste krekel, ik ga naar de stad **23.** van de koning. Ik wil de prinses **24.** met het treurige gezicht aan het **25.** lachen maken en het grote witte **26.** paard IJzerbreker beslaan. Zo zal **27.** ik de schoonzoon en erfgenaam **28.** van de koning worden.""Kri, kri, kri, **29.** beste smid, neem me mee, dan kan ik je **30.** misschien nog van dienst zijn." **31.** "Met alle plezier, mijn beste krekel. **32.** Klamp je maar stevig vast aan mijn kin." **33.** De smid zette zijn weg verder met **34.** de krekel stevig aan zijn kin. Drie **35.** uur later zat hij opnieuw aan **36.** de kant van de weg om uit te rusten **37.** en wat te eten en te drinken. **38.** "Piep, piep, piep, gegroet, beste smid, **39.** "riep een rat die in een nabijgelegen **40.** akker aan een tabaksblad knabbelde. **41.** "Jij ook gegroet, mijn beste rat. **42.** Waarmee kan ik je van dienst zijn?" **43.** "Piep, piep, piep, beste smid, ik wil **44.** alleen maar weten waar je heen gaat." **45.** "Mijn beste rat, ik ga naar de stad **46.** van de koning. Ik wil de prinses met **47.** het treurige gezicht aan het lachen **48.** maken en het grote witte paard **49.** IJzerbreker beslaan. Zo zal ik de schoonzoon **50.** en erfgenaam van de koning worden." **51.** "Piep, piep, piep, beste smid, neem **52.** me mee, dan kan ik je misschien nog **53.** van dienst zijn." "Met alle plezier, mijn **54.** beste rat, klamp je maar stevig vast aan **55.** mijn muts. "De smid vervolgde zijn weg **56.** met de krekel aan zijn kin en de rat op **57.** zijn muts. Die avond snurkte hij tevreden **58.** in een herberg, maar bij het aanbreken **59.** van de ochtend werd hij met een schok wakker **60.** door een beet in het puntje van zijn neus.

Comprehension Questions

Block 1: *Het verhaal gaat over een koning die onmetelijk rijk is, zo vrijgevig als een priester, zo dapper als een leeuw en zo eerlijk als goud. (Y); De koning was erg gelukkig. (N); De dochter van de koning werd 'de prinses met het treurige gezicht' genoemd. (Y); Het enige paard waar de koning van hield was zwart. (N); Het grote witte paard was zo'n goedaardig beest dat zelfs de slechtste smid ter wereld al zijn hoeven kon beslaan. (N); Het grote witte paard werd 'IJzerbreker' genoemd. (Y); De man die de prinses aan het lachen kon maken en het favoriete paard aan vier hoeven kon beslaan zou de schoonzoon en de erfgenaam van de koning worden. (Y); Een kleine hoeveelheid kandidaten die hun geluk wilden beproeven, meldde zich aan. (N); Een flinke jonge smid probeerde om de schoonzoon en erfgenaam van de koning te worden. (Y); Het hele vermogen van de jonge smid was 100 zilverstukken en 50 goudstukken. (Y)*

Block 2: *Van de 100 zilverstukken smeedde de smid 4 zilveren spijkers. (N); Van de 50 goudstukken smeedde de smid 28 gouden hoefijzers. (N); De smid ging met een leren tas met een brood, een fles wijn, een hamer, de hoefijzers en de spijkers naar de stad van de koning. (Y); Onderweg kwam de smid in een nabijgelegen korenveld een kraai tegen die zo zwart was als roet uit een schoorsteen. (N); Een krekel vroeg aan de smid of hij hem mee kon nemen zodat deze de smid misschien nog van dienst kon zijn. (Y); De smid zette zijn weg voort met de krekel stevig aan zijn kin. (Y); Toen de smid aan het uitrusten was kwam hij een rat tegen die in een nabijgelegen vuilnisbak aan een rotte banaan knabbelde. (N); De smid nam de rat mee naar de stad van de koning, zodat deze hem misschien nog van dienst kon zijn. (Y); De smid vervolgde zijn weg met de krekel aan zijn kin en de rat in zijn jas. (N); De smid werd met een schok wakker door een beet in het puntje van zijn kin. (N)*

Van Drie Slimme Dieven

Block 1

1. Drie dieven zwierven eens door het **2.** bos, in de hoop dat er iemand langs het **3.** pad zou komen, die ze konden bestelen. **4.** Daar kwam een boer aan op een ezeltje, **5.** een geit met zich meevoerend aan een lang **6.** touw. Vrolijk klingelden de belletjes aan de **7.** halsband van de geit en de boer, die **8.** een stevig glaasje wijn had gedronken, zong **9.** een liedje op de maat van dat geklingel. **10.** "Kijk die geit eens!" fluisterde de ene dief **11.** tegen zijn kameraden, "wat een pracht **12.** van een beest!" "Nou maar, dat ezeltje **13.** mag er ook wezen, hoor!" zei de tweede. **14.** "En ik moet aldoor kijken naar de mooie **15.** nieuwe kiel, die die pummel aan heeft!" **16.** riep de derde. "Zeg jongens, we moesten **17.** eens proberen hem zijn geit, zijn ezel en**18.** zijn kiel te ontstelen! Als we dat klaarspelen, **19.** mogen we met recht knappe dieven heten!" **20.** "Ja, ja, dat moeten we proberen! Ik neem **21.** de geit voor mijn rekening!" zei de eerste **22.** weer. "En als ik dan de ezel steel en jij **23.** de kiel, dan is het zaakje gezond," lachte **24.** de tweede tegen de derde. Zo gezegd, zo **25.** gedaan! Nummer één zou eerst zijn slag slaan, **26.** en ondertussen verstopten de twee

anderen **27.** zich in het struikgewas. En de boer reed **28.** maar voort, aldoor 't hoogste lied zingend **29.** bij 't klingelen van de geitenklokjes. Geen **30.** ogenblik kwam hij op de gedachte, dat er **31.** wel eens dieven op hem zouden kunnen **32.** loeren in dit dichte bos. Jan - zo heette de **33.** eerste dief - sloop nu stilletjes uit de **34.** struiken te voorschijn en ging naast de geit **35.** lopen, op de maat van het gezang van **36.** de boer. Hij streelde haar telkens, krauwde **37.** haar tussen de horens en - maakte onder **38.** de hand haar halsband met belletjes **39.** voorzichtig los en bond die aan de staart **40.** van de ezel. Daarna sneed hij vlug het **41.** touw door, verdween met de geit in het **42.** bos en bond haar daar aan een boom, **43.** terwijl de ezel nu de belletjes vrolijk liet **44.** klinken, telkens als hij met zijn staart **45.** naar de vliegen sloeg, die hem plaagden. **46.** En de boer zong maar en zong maar het **47.** ene liedje na het andere, op de maat **48.** van 't belletjesgeklingel. Maar eindelijk **49.** keek hij toch eens even om - en o wee! **50.** - daar merkte hij dat zijn geit verdwenen **51.** was! "Hè," riep hij verbaasd, "waar is toch **52.** mijn mooie geit gebleven? Ze heeft **53.** zeker het touw doorgebeten en is er **54.** op haar eentje vandoor gegaan!" Meteen **55.** sprong hij van zijn ezel, liet het beest **56.** aan de weg staan bij een troep distels, **57.** waar het dadelijk van begon te smullen, **58.** en liep een eind terug, overal goed **59.** rondkijkend en zijn sikje roepend. Maar **60.** hoe hij ook zocht, hoe vleiend hij ook:

Block 2
1. "Sik, Sik! Sik, Sikkie!" riep, de geit was **2.** weg en bleef weg. Ja, dan moest hij het **3.** maar opgeven! Zuchtend liep hij terug **4.** naar de plek, waar hij zijn ezel had gelaten, **5.** maar - wat was dat? - daar was me die **6.** waarlijk ook al verdwenen! Terwijl hij **7.** nu stond te vloeken en te lamenteren op **8.** de weg, kwam daar juist een man voorbij, **9.** die hem vol medelijden vroeg, wat **10.** hem scheelde? Deze man was niemand **11.** anders dan de derde dief, maar dat wist **12.** die arme boer natuurlijk niet. "Wat mij **13.** scheelt?" jammerde de boer, "ik ben van **14.** huis gegaan met een prachtige geit en **15.** de mooiste ezel van de hele wereld **16.** - en nu ben ik ze allebei kwijt geraakt op **17.** deze vervloekte weg!" "Ach, ach, arme **18.** man! Nee, maar, dat is verschrikkelijk! **19.** Maar ja, nu ik me goed bedenk, schiet **20.** het me opeens te binnen, dat ik zopas **21.** een ezel in een put heb zien vallen. Kan **22.** dat de jouwe misschien ook geweest zijn?" **23.** "Waar is die put? Zeg 't mij gauw! Ik **24.** moet er dadelijk naar toe!" "O, heel **25.** dichtbij. Wacht, ik zal je wel eventjes de **26.** weg wijzen." Een ogenblik daarna stonden **27.** ze samen bij de put. "Luister!" riep opeens **28.** de dief. "Wat was dat? 't Was net of ik **29.** iemand hoorde roepen van uit de diepte! **30.** " En hij boog zich over de put en riep: "Jan! **31.** Jan!" Dadelijk antwoordde de echo: "Han! **32.** Han!" "Vreemd," zei de dief, "zou 't heus **33.** je ezel kunnen zijn, die de baas roept?" **34.** "Ja, ja, dat moet mijn lieve Grauwtje zijn!" **35.** riep de boer opgewonden. "Zeg, help me **36.** even, dan ga ik hem halen! Heb je toevallig **37.** ook een stuk touw bij je?" Ja, dat had de **38.** dief wel, en hij wou het de boer ook wel **39.** om zijn middel binden en hem voorzichtig **40.** in de put laten afzakken. "Maar zeg, mijn **41.** goeie man," riep hij opeens, alsof hem **42.** plotseling iets in de zin kwam, "zou **43.** je niet liever eerst je kleren uittrekken? **44.** Anders worden die immers door en door **45.** nat. Geen sterveling ziet je hier. Je kunt **46.** het gerust wagen!" "Wel ja," riep de boer, **47.** "dat heb je goed bedacht! Man man, wat **48.** ben ik blij dat ik jou ontmoet heb! **49.** Anders had ik me geen raad geweten! **50.** Ziezo, en laat me nu maar zakken!" Hij **51.** was nu geheel naakt en de dief liet **52.** hem voorzichtig neer in de put. Maar **53.** toen de arme kerel beneden was – de **54.** put was gelukkig droog! - liet de ander **55.** het touw los en floot zijn kameraden, **56.** die vol spanning tussen het kreupelhout **57.** lagen af te wachten, hoe dit zaakje zou **58.** aflopen. Ziezo, dat hadden ze nu alle drie **59.** eens netjes opgeknapt! Daar konden ze **60.** trots op zijn en - daar moesten ze allereerst

Comprehension Questions

Block 1: *Het verhaal gaat over drie dieven die door het bos zwierven. (N); Er kwam een boer aan op een paard, een geit met zich meevoerend aan een lang touw. (N); De boer had een stevig glaasje wijn gedronken. (Y); De dieven vonden dat ze eens moesten proberen om de boer zijn geit, zijn ezel en zijn paard te ontstelen. (N); De eerste dief zou eerst zijn slag slaan, terwijl de twee andere dieven zich gingen verstoppen in het struikgewas. (Y); De eerste dief sloop nu stilletjes uit de struiken tevoorschijn en ging naast het paard lopen, op de maat van het gezang van de boer. (N); De eerste dief maakte de halsband met belletjes voorzichtig los en bond die aan de staart van de ezel. (Y); De eerste dief sneed vlug het touw door, verdween met het paard in het bos en bond haar daar aan een boom, terwijl de ezel nu de belletjes vrolijk liet klinken. (N); De boer keek eindelijk toch eens even om – en o wee! – daar merkte hij dat zijn paard verdwenen was! (N); De boer sprong van zijn ezel en liet het beest aan de weg staan bij een troep distels. (Y)*

Block 2: *De geit was weg en bleef weg. (Y); Zuchtend liep de boer terug naar de plek waar hij zijn ezel had achtergelaten. (Y); De man die langskwam was niemand anders dan de derde dief. (Y); De boer jammerde dat hij van huis was gegaan met een prachtig paard en de mooiste kip van de hele wereld en dat hij ze nu allebei was kwijtgeraakt op deze vervloekte weg. (N); De derde dief zei dat hij zopas een ezel in de put had zien vallen. (Y); De dief wees de boer de weg naar de put. (Y); De dief had geen stuk touw bij zich. (N); De dief vroeg of de boer niet liever eerst zijn kleren uit wilde trekken. (Y); De boer was niet blij dat hij de dief ontmoet had. (N); De dief liet het touw los en floot zijn kameraden, die vol spanning tussen het kreupelhout lagen af te wachten. (Y)*

Dutch – Serbian Condition

De Rode Schoentjes

Block 1

1. Er was eens een klein meisje, **2.** dat heel mooi en allersnoezigst **3.** was. 's Zomers moest ze altijd **4.** op blote voeten lopen, want ze **5.** was arm, en 's winters op grote **6.** klompen, zodat haar wreef helemaal **7.** rood werd. Heel akelig was dat. **8.** Midden in het boerendorp woonde **9.** de oude schoenmakersvrouw. **10.** Ze zat zo goed als ze kon van oude, **11.** rode stofrestjes een paar schoentjes **12.** te maken, heel prutserig, maar **13.** het was goed bedoeld. Die schoentjes **14.** waren voor het kleine meisje. **15.** Dat kleine meisje heette Karen. **16.** Precies op de dag dat haar moeder **17.** werd begraven, kreeg ze de rode **18.** schoentjes en had ze voor het **19.** eerst aan. Eigenlijk was het niet iets **20.** om te dragen als je in de rouw bent, **21.** maar ze had nu eenmaal geen **22.** andere en dus liep ze met blote **23.** benen in de rode schoentjes, **24.** achter de armzalige kist van geperst **25.** stro. Op hetzelfde moment kwam **26.** er een grote, oude koets aan **27.** en daar zat een oude mevrouw in. **28.** Ze keek naar het kleine meisje en **29.** ze had medelijden met haar, **30.** en dus zei ze tegen de dominee: **31.** "Luister, geef mij dat kleine **32.** meisje, dan zal ik goed voor haar zorgen." **33.** Karen dacht dat het allemaal **34.** door de rode schoentjes kwam, **35.** maar de oude mevrouw zei dat die **36.** vreselijk waren. Ze werden verbrand, **37.** en Karen zelf kreeg keurige kleertjes **38.** aan. Ze moest leren lezen en naaien **39.** en de mensen zeiden dat ze er **40.** snoezig uitzag, maar de spiegel zei: **41.** "Je bent meer dan snoezig. Je bent mooi!" **42.** Toen reisde de koningin op een **43.** keer door het land en ze had haar **44.** dochtertje, die een prinses was, **45.** bij zich. De mensen stroomden toe **46.** vóór het kasteel en Karen was er ook bij. **47.** Het kleine prinsesje stond in mooie, **48.** witte kleertjes voor het raam en **49.** liet zich bekijken. Ze had geen **50.** sleep en ook geen gouden kroontje, **51.** maar ze had mooie, rode, lakleren **52.** schoentjes aan. Dat was wel even **53.** iets anders dan de schoentjes die de **54.** schoenmakersvrouw voor Karen had **55.** gemaakt. Niets in de wereld is met die **56.** rode schoentjes te vergelijken. **57.** Karen was nu oud genoeg om naar **58.** de kerk te gaan. Ze kreeg nieuwe **59.** kleren en nieuwe schoenen **60.** moest ze ook hebben. De rijke

Block 2

1. schoenmaker in de stad nam **2.** de maat van haar voetje, thuis **3.** in zijn eigen kamer. Daar stonden **4.** grote glazen kasten met beeldige **5.** schoentjes en met glimmende laarzen. **6.** Het zag er prachtig uit, maar de oude **7.** mevrouw kon niet meer zo goed zien, **8.** dus veel plezier had ze er niet van. **9.** Midden tussen de andere schoenen **10.** stond ook een paar rode schoentjes, **11.** precies als die van de prinses. **12.** Mooi dat ze waren! De schoenmaker **13.** zei dan ook dat ze voor de dochter **14.** van een graaf bestemd waren, **15.** maar dat ze haar uiteindelijk niet pasten. **16.** "Dat zijn zeker lakschoentjes," zei de oude **17.** mevrouw. "Ze glimmen zo." - "Ja, ze **18.** glimmen," zei Karen. Ze pasten en ze **19.** werden gekocht, maar de oude **20.** mevrouw wist niet dat ze rood **21.** waren, want ze had het nooit **22.** goed gevonden dat Karen naar **23.** de kerk ging met rode schoenen **24.** aan haar voeten, maar dat gebeurde **25.** dus wel. Alle mensen keken naar haar **26.** voeten en toen ze over het middenpad **27.** naar het koor liep, dacht ze dat zelfs **28.** de oude schilderijtjes op de graven, **29.** die portretten van dominees en **30.** domineesvrouwen, met stijve kragen **31.** en lange, zwarte toga's, hun ogen op **32.** haar rode schoentjes richtten. Ze dacht **33.** nergens anders aan toen de dominee **34.** zijn hand op haar hoofd legde **35.** en over het heilig doopsel sprak, **36.** over het verbond met God en **37.** dat ze nu een volwassen christen **38.** zou zijn. Het orgel speelde heel plechtig, **39.** de mooie kinderstemmetjes zongen **40.** en de oude voorzanger zong, maar Karen **41.** dacht alleen aan de rode schoentjes. **42.** 's Middags had de oude mevrouw **43.** intussen van alle mensen gehoord **44.** dat de schoentjes rood waren en **45.** ze zei dat dat lelijk was, dat het niet **46.** hoorde en dat Karen van nu af aan, **47.** als ze naar de kerk ging, altijd zwarte **48.** schoenen aan moest, ook al **49.** waren het oude schoenen. **50.** De volgende zondag was het **51.** Avondmaal en Karen keek naar **52.** de rode schoentjes, ze keek naar **53.** de zwarte - en toen keek ze weer **54.** naar de rode en trok de rode aan. **55.** De zon scheen heerlijk. Karen en **56.** de oude mevrouw liepen over het **57.** pad door het koren, waar het een **58.** beetje stoffig was. Bij de kerkdeur stond **59.** een oude soldaat met een kruk als stok **60.** en met een wonderlijke lange baard, eerder

Comprehension Questions

Block 1: *Het kleine meisje liep in de zomer op blote voeten omdat ze geen schoentjes had. (Y); De oude schoenmakersvrouw woonde midden in het boerendorp. (Y); De oude schoenmakersvrouw maakte hele mooie schoentjes. (N); De oude schoenmakersvrouw maakte schoentjes voor de verjaardag van het meisje.(N); Het kleine meisje droeg de rode schoentjes naar de begrafenis van haar moeder omdat ze geen andere schoentjes had. (Y); Een oude mevrouw in een koets wilde voor het meisje zorgen omdat zij de rode schoentjes mooi vond. (N); Het kleine meisje kreeg nette kleren van de oude mevrouw. (Y); Het meisje Karen was een prinsesje en woonde bij de koningin. (N); Het kleine prinsesje had rode lakleren schoentjes.(Y); De rode schoentjes van Karen waren zo mooi als de lakleren schoentje van de prinses.(N)*

Block 2: *Het kleine meisje kreeg nieuwe schoenen van een arme schoenmaker. (N); Het kleine meisje kwam bij de schoenmaker thuis om de maat van haar voetjes op te laten nemen. (Y); De oude mevrouw bekeek het huis van de schoenmaker en vond het erg mooi. (N); De schoenmaker had rode schoentjes, net zoals die van de prinses. (Y); De schoenmaker had de rode schoentjes speciaal gemaakt voor het kleine meisje Karen. (N); De oude mevrouw wist*

dat het kleine meisje rode schoentjes kreeg. (N); Karen ging naar de kerk met haar rode schoentjes en werd door iedereen bekeken. (Y); Karen luisterde aandachtig naar het orgel en het koor in de kerk.(N); De oude mevrouw wilde dat Karen zwarte schoentjes aan zou doen naar de kerk, ook al waren dat oude schoentjes. (Y); De volgende zondag droeg Karen de zwarte schoentjes naar de kerk, zoals de oude mevrouw dat wilde.(N)

Bajka o Jasenki i Rabasku (Serbian)

Block 1

1. Teče reka Rabas između brda, **2.** šuma, livada i njiva. Žubori, šumi, **3.** negde utihne, na nekom mestu kao da se **4.** njegova voda svađa sa kamenjem koje je **5.** prekrilo dno. Sunce se probija kroz **6.** lišće drveća i, evo - već nekoliko **7.** najupornijih zraka uronilo je u rečicu, **8.** pozlatilo ribice i račiće i prozirna krila **9.** vilinog konjica koji je leteo nad vodom. **10.** Videli ljudi da je dobro imati put pored reke **11.** pa ga i napravili. Prolaze putem kad idu **12.** u vodenicu, u susedno selo poslom ili na veselje **13.** a i da se nađu sa prijateljima u teškim trenucima. **14.** Putem ide i daždevnjak u svom najlepšem **15.** crno-žutom odelu, a i zmija vijuga **16.** žureći u kamenjar. Tu su i srne koje **17.** sa srndaćem prelaze put da bi otišle **18.** u drugu šumu. Zastanu na putu, **19.** osluškuju nešto,onjuše, zatrepću **20.** svojim krupnim očima, iz kojih **21.** kao da će svakog trenutka kanuti suze, **22.** i odlaze dalje. Šuma kraj puta sanja **23.** i smeši se u snu. Ptice se ljuljuškaju **24.** na grani. Šumske životinje se pritajile. **25.** Samo leptir velikih šarenih krila, **26.** nečujno lebdi iznad cvetova kao da **27.** traži nešto. Dunu vetrić. Šuma se **28.** trže iz sna, životinjice se pokrenuše, **29.** leptir pronađe cvet koji je tražio **30.** i sklopi krila. Vetar nešto šapnu šumi, **31.** a ona zatrese lišćem. Ptice zacvrkutaše **32.** na granama. Ču se žamor. Od cveta **33.** do cveta, od lista do lista, od grane do grane: **34.** "Šumska vila rodila kćerku! Šumska vila **35.** rodila kćerku!" Orilo se hiljadu glasića šumom. **36.** Pevalo se, veselilo. Dolete slavuj **37.** sa svojom porodicom i kad ču za novost, **38.** upita: "Kako se zove?" Tad se setiše **39.** da maloj vili treba dati ime. **40.** Beli jasenov cvet šapnu grani **41.** "Neka se zove Jasenka, jer je **42.** ovde rođena." Šapat se pretvorio u žamor. **43.** Svima bi po volji: Jasenka je **44.** lepo ime. Teče reka Rabas. Ribe, **45.** i velike i male, plivaju, iskaču **46.** iz vode, igraju se, talasići govore nešto **47.** jedan drugom, a žalosna vrba spustila grane **48.** do same vode da čuje kakvu to tajnu **49.** talasići imaju. I čula je: "Rodio se **50.** vodeni duh! Rodio se vodeni duh!" **51.** Radoznala žalosna vrba zatrese **52.** svojom dugom zelenom kosom i upita: **53.** "A kako se zove beba?" Rabas **54.** ponosno zažubori: "Rabasko, draga vrbo, **55.** Rabasko, tako sam srećan!" Vrba **56.** ništa ne odgovori, već uzdahnu a jato ptica **57.** polete. Tako se i u šumi i u reci proslavljalo **58.** zbog dve bebe: Jasenke i Rabaska. **59.** Raste mala šumska vila Jasenka. **60.** Raste i raduje se svemu što vidi, čuje, oseti.

Block 2

1. Razigrana, raspevana, omiljena. **2.** Peva sa pticama i cvrčcima, plače **3.** Sa malom bubamarom koja se izgubila. **4.** Pomaže pauku da isplete mrežu. **5.** Pentra se uz stabljiku šumskog cveta **6.** da pozdravi pčelu koja iz polena siše **7.** slatki sok. Bezbrižnu, nosi je leptir **8.** kad joj se prohte da leti. Iz prikrajka **9.** prate je nečije oči mračne i stroge. **10.** To je šumski vilenjak. Smeška se zlurado. **11.** Ne ume drugačije. Ne zna da se raduje. **12.** Svi ga se plaše i izbegavaju ga. **13.** On to zna i drago mu je što je tako. **14.** Sam, neuznemiravan. Samo da nije **15.** onih brbljivih ptica koje su savile gnezdo **16.** u blizini njegove pećine. Cvrkuću o ljubavi. **17.** Pih: ljubav! Važna je samo moć. **18.** Tako razmišlja zli vilenjak i čeka **19.** da Jasenka poraste. Uzeće je za ženu. **20.** Da Jasenka ne bi pristala, o tome **21.** ne misli, jer ko bi smeo i da pomisli **22.** da se suprotstavi njemu,koji je najstrašnije **23.** biće u šumi. Trudiće se da ona, **24.** kad postane njegova žena, bude **25.** okrutna kao i on. I zla, bez osmeha. **26.** To će biti prava stvar. Ovakva kakva je **27.** nije ono što on želi. Video je kad je **28.** pomagala jednoj maloj ljubičici da **29.** otvori latice. Dobrota je najveća glupost. **30.** A on,prepun mržnje i zla izlečiće je **31.** od dobrote i plemenitosti. Sa takvim mislima, **32.** zli vilenjak se povuče u tamu svoje pećine. **33.** Rabasko je bio nestašan vodeni duh. **34.** Skakao je sa kamena na kamen, **35.** sa grane a granu, sa grane u vodu. **36.** Plašio je ribe i račiće, oponašao **37.** žubor vode kad bi Rabas presušio **38.** pa su ljudi prolazeći putem, u čudu **39.** vrteli glavom i krstili se. Topao dan. **40.** Ni daška vetra, a lišće na vrbama **41.** treperi. To Rabasko plete vrbinu **42.** zelenu kosu. Smenjivali se dani i noći. **43.** Smenjivala se godišnja doba. **44.** Iza duge zime dođe proleće. **45.** Jasenka je trčala šumom. Sneg **46.** se topio i prvo prolećno cveće **47.** je pozdravljalo malu šumsku vilu. **48.** Jasenka, razdragana doskakuta do ivice šume. **49.** Rabasko se baš ljuljuškao na vrbinom listu, **50.** kad ugleda Jasenku. Iznenađen i ushićen, **51.** on kliznu sa vrbinog lista u Rabas. **52.** Talasići se nasmejaše, a ribe se zagnjuriše u vodu. **53.** Rabasko još jednom pogleda, protrlja oči, **54.** ali Jasenka je još uvek bila na ivici šume. **55.** I njeno srce je brzo, brzo kucalo, **56.** a znala je i zašto: zaljubila se. Poželela je **57.** da poljubi svaki cvetić, svaku travčicu. **58.** "Ovo je žena mog života!" pomisli Rabasko. **59.** "Sve ću učiniti da bude moja zauvek!" **60.** Šuma je znala a znao je i Rabas da je sve uzalud.

De Twee Muilen

Block 1

1. Er was eens een bejaarde koopman, **2.** Aboe Kasim heette hij, die de rijkste **3.** koopman in heel Perzië was. Hij was **4.** overigens zo gierig, dat hij in lompen **5.** door de straten van de stad liep, alsof **6.** hij een arme bedelaar was. Zijn gewaad **7.** was oud en tot op de draad versleten **8.** en zo vaak versteld met lappen in **9.** andere kleuren, dat niemand meer kon **10.** beoordelen welke kleur het kledingstuk **11.** oorspronkelijk had gehad. Met zijn tulband **12.** stond het al niet veel beter. Het was een **13.** groezelige tulband van de goedkoopste **14.** stof, die ooit op enige markt was **15.** verkocht en die meer aan een dweil voor **16.** het schoonmaken van vloeren deed **17.** denken dan aan een

hoofdtooi. **18.** Dit alles werd nog ver overtroffen door **19.** zijn muilen. In de zolen zaten grote **20.** gaten en de bovenkanten vertoonden een **21.** mozaïek van opgezette lapjes - zoveel, **22.** dat men bij het tellen in de war zou zijn **23.** geraakt. Zeker tien jaar lang hadden **24.** diverse schoenlappers hun beste krachten **25.** aan die haveloze muilen gewijd en **26.** het was hun steeds moeilijker gevallen ze **27.** bijeen te houden. De muilen waren **28.** geleidelijk zo berucht geworden, dat **29.** ieder die wilde beschrijven dat iets totaal **30.** waanzinnig of onmogelijk was, aan zijn **31.** woorden toevoegde: "alsof het de **32.** muilen van Kasim waren!" Op een avond **33.** liep de koopman in voortreffelijk **34.** humeur over de markt. Hij had die **35.** ochtend een grote partij geslepen **36.** drinkglazen kunnen kopen tegen een **37.** zacht prijsje, doordat de man die ze **38.** te koop aanbood, hals over kop naar **39.** zijn glasblazerij terug moest en geen **40.** tijd voor onderhandelen had. Tegen **41.** de middag was hij aangeschoten door **42.** iemand die op zwart zaad zat, zijn **43.** schulden niet kon betalen en Kasim het **44.** enige te koop aanbood dat hij nog **45.** bezat - een partij rozenolie voor de halve **46.** prijs. Volgens gebruik dienden dergelijke **47.** voordelige zaken gevierd te worden, men **48.** nodigde dan zijn vrienden uit voor een **49.** feestmaal. Kasim evenwel was van **50.** mening beter naar de badinrichting te **51.** kunnen gaan. Hoewel hij op water even **52.** gek was als de kat, viel het niet te **53.** ontkennen dat hij in het badhuis goedkoper **54.** uit zou zijn dan thuis met zijn vrienden, **55.** die hem de oren van het hoofd zouden **56.** eten. Terwijl hij zich in de kleedkamer van **57.** zijn plunje ontdeed en juist uit zijn **58.** muilen wilde stappen, werd de koopman **59.** Op de schouder getikt door een van **60.** zijn buren, met wie hij tamelijk goed

Block 2

1. bevriend was. "Die muilen van jou, **2.** daar maakt de hele stad zich vrolijk **3.** om, Kasim, " zei de vriend. "Het **4.** wordt hoog tijd dat je een paar nieuwe **5.** koopt. Geloof me, iedereen steekt de **6.** draak met je." "Daar heb ik al heel **7.** lang over gedacht," antwoordde de **8.** koopman en hij kreeg rimpels in zijn **9.** voorhoofd; "ik heb voor en tegen van zo'n **10.** aankoop langdurig overwogen. Ik ben **11.** jammer genoeg nog niet tot een besluit **12.** gekomen, ik weet niet of mijn muilen **13.** werkelijk zo versleten zijn, dat ze niet **14.** meer gebruikt kunnen worden." Kasim **15.** had niet eens gemerkt dat ze onder **16.** het praatje tot vlak bij de rand van **17.** het bad waren gekomen. Klappertandend **18.** stak hij behoedzaam de grote teen van **19.** zijn rechtervoet in het water, alsof hij **20.** zich in de bek van een leeuw waagde. **21.** Terwijl hij zich kweet van de hoogst **22.** onaangename wastaak, werd de deur **23.** aan de straat geopend en de eerste **24.** rechter van de stad kwam binnen, ook **25.** met het doel een bad te nemen. De **26.** koopman was al heel gauw klaar en **27.** repte zich naar de kleedkamer, waar **28.** hij zijn bundeltje kleren bijeen zocht **29.** en zich haastig aankleedde. Maar bij **30.** de baard van de Profeet, waar waren **31.** zijn oude muilen? Waar hij ze achtergelaten **32.** had, stond nu een paar fraaie nieuwe **33.** pantoffels. Hij nam aan dat ze een **34.** geschenk waren van de buurman, met **35.** wie hij even tevoren had gepraat. **36.** Zonder zich te bedenken stak de oude **37.** vrek er zijn voeten in. "Aardige man," **38.** dacht hij bij zichzelf, "hij heeft me de **39.** aanschaf kosten van een paar nieuwe **40.** muilen willen besparen." Opgeruimd **41.** verliet hij de badinrichting. Onderwijl **42.** was ook de rechter klaar met zijn bad. **43.** Hij kleedde zich op zijn gemak aan, **44.** maar toen hij zijn voeten in zijn pantoffels **45.** wilden steken, bleken ze verdwenen en **46.** vruchteloos zocht de badknecht naar **47.** het fraaie schoeisel met het goudborduursel. **48.** In een hoek van de kleedkamer vond **49.** men alleen de opgelapte muilen met **50.** de losse zolen, die de rechter dadelijk **51.** als het eigendom van Kasim herkende. **52.** "Dacht je zo van je oude muilen af te **53.** komen, jij schurk?" mompelde de rechter. **54.** "Je bent niet alleen een dief, maar een **55.** brutale rakker ook. Je moet wel lef hebben, **56.** als je de eerste rechter van de stad gaat **57.** bestelen, maar je zult ervan lusten, vriend!" **58.** De rechter stuurde zijn sterkste knecht **59.** naar het huis van de vrek en Kasim werd **60.** al gauw voor hem geleid. Om te beginnen

Comprehension Questions

Block 1: *Het verhaal gaat over een bejaarde koopman, Aboe Kasim, die de rijkste koopman in heel Perzië was. (Y); Aboe Kasim was vrijgevig. (N); Het gewaad van Aboe Kasim was nieuw en heel erg mooi. (N); De groezelige tulband van Aboe Kasim was van de goedkoopste stof. (Y); In de zolen van de muilen van Aboe Kasim zaten grote gaten. (Y); Slechts 10 dagen hadden diverse schoenlappers hun beste krachten aan die haveloze muilen gewijd. (N); Op een avond liep de koopman in een slecht humeur over de markt. (N); De man moest terug naar de bierbrouwerij. (N); Het enige dat Aboe Kasim te koop had was rozenolie. (Y); De Koopman werd op de schouder getikt door zijn vrouw. (N)*

Block 2: *Aboe Kasim's muilen maakten de hele stad vrolijk. (Y); Aboe Kasim overwoog niet om nieuwe muilen te kopen. (N); Ze waren tijdens het praatje tot vlak bij de glasblazerij gekomen. (N); Het water van het bad was warm. (N); De eerste rechter van de stad kwam binnen door de deur aan de straat. (Y); De rechter wilde ook een bad nemen. (Y); De koopman was zijn muilen kwijt. (Y); De rechter was nog lang niet klaar met zijn bad. (N); De rechter kon zijn fraaie schoeisel niet meer vinden. (Y); De opgelapte muilen met de losse zolen herkende de rechter als het eigendom van Aboe Kasim. (Y)*

Mače Moni i mišić Boni (Serbian)

Block 1

1. Jasna je išla iz škole.Ranac na leđima **2.** bio joj je težak. Išla je lagano i razmišljala **3.** kako to da danas, onako lak zadatak **4.** iz matematike nije uspela da reši. **5.** Ide Jasna, gleda u vrhove svojih patika **6.** i vidi nešto – malo i žuto mače. **7.** "Ne na ulicu!" kaže Jasna, a mače **8.** gleda u Jasnu i mjauče jedva čujno. **9.** "Ti si maco gladna" kaže Jasna i

10. vadi iz ranca kesu sa polupojedenom užinom. **11.** Mače halapljivo pojede ponuđenu užinu **12.** i, evo, već prede zadovoljno. Te večeri **13.** Jasna je unela mače tako da roditelji **14.** nisu primetili. "Baš si ti umiljata maca" **15.** kaže Jasna, a mače trlja glavicu o Jasninu nogu. **16.** "Kako da te zovem? pita Jasna macu **17.** ali izbor imena je, naravno, njen zadatak. **18.** Posle dužeg razmišljanja, odluči da će se **19.** mače zvati Moni.Sad je pola posla **20.** bilo gotovo.Ujutro će reći mami kako je **21.** mače bilo u njihovom vrtu. Mama voli životinje **22.** ali nije raspoložena da ih gleda **23.** kako šetaju po kući. Valjda će nekako **24.** uspeti da odobrovolji majku. Kada je **25.** sledećeg jutra Jasna sišla na doručak, **26.** čula je kako mama iznervirano govori tati **27.** "Zamisli, Bane, imamo miša u kuhinji! **28.** Pa to je strašno! Otkud on?! I to neki mali, **29.** ovolicki (pokazuje mužu od palca do kažiprsta)". **30.** "A imamo i mače" nadovezuje se Jasna, **31.** jer misli da je ovo pravi trenutak **32.** "ovolicko" (i ona pokazuje koliko je mače **33.** približavajući obe šake jednu drugoj **34.** kao da će da aplaudira). Tata poče **35.** da se smeje. "Pa ovaj dan je **36.** dobro počeo: imamo dva gosta!" **37.** Mama gleda u Jasnu koja, preskačući **38.** po dve stepenice, ode u svoju sobu **39.** i donese Monija. "E, draga, sad ti idi **40.** u kuhinju i donesi svog maleckog miša!" **41.** nastavlja tata šalu. Mama ćuti, ne zna **42.** da li da nastavi da se nervira zbog miša ili **43.** što je mače provelo noć u Jasninoj sobi. **44.** Jasna uzme šolju za kafu bez drške, **45.** usu malo mleka iz svoje šolje **46.** i stavi ispred Monija. Najzad, mama upita: **47.** "Otkud ti mače?" Ali tata opet **48.** spasi situaciju rekavši: "Ženo, **49.** danas spremaš ručak za petoro." **50.** Mama ne reče ništa, ustade i poče **51.** da sklanja ostatke od doručka. **52.** Jasna je sijala od sreće: mama je **53.** neće grditi, jer je tata, mada u šali, **54.** dao dozvolu da njen kućni ljubimac **55.** ostane u kući. Mišić se pojavi u **56.** trpezariji ali ga niko ne primeti, **57.** osim Monija. "Uzmi malo!" reče **58.** tiho Moni. "Ja sam mišić Boni i **59.** ne mogu s tobom da pijem mleko **60.** jer ti možeš da me pojedeš."

Block 2
1. "Neću, časna reč. Da sam hteo **2.** da te pojedem, mogao sam to odmah **3.** da uradim, a ne da s tobom pričam, **4.** zar ne?" "Biće da je tako" reče **5.** bojažljivo Boni, lagano se približavajući **6.** šoljici s mlekom. "Ja sam mače Moni **7.** i miševi mi nisu na jelovniku. **8.** Čuo sam od bake da smo nekad **9.** jeli miševe ali to je davna prošlost." **10.** Boni priđe i poče da pije mleko. **11.** "Hvala ti drugar, baš si kul! **12.** Ti si iz ove kuće, zar ne?" **13.** "Ne" reče Moni "donela me Jasna sinoć, **14.** a ti?" "Ne znam šta da ti kažem. **15.** Sinoć smo moji roditelji, braća,sestre i ja **16.** večerali. Onda odjednom tata viknu: Beži! **17.** I ja sam bežao, bežao, bežao…"**18.** "Stani!" reče Moni. "Stao sam" uzbuđeno **19.** reče Boni "i jutros ja kod nekog šporeta, **20.** a žena viče:'Ju, ju, miš !' Otkud ja u kući, **21.** otkud kod šporeta – pojma nemam! **22.** Čudo! Kažem ti. Kakva je to kuća, **23.** nigde rupe da se zavučem u nju! I eto, **24.** sad pijem mleko sa mačkom! To je san!" **25.** "Ma, smiri se, Boni, ne sanjaš! Pij mleko!" **26.** "Kaži šta ja da radim, gde da idem, **27.** ne znam!" "Ne brini, Boni, sve će to **28.** ispasti dobro. Eto, živećeš ovde sa mnom.." **29.** "Jel?! Baš si duhovit, Moni! " **30.** Tebe će devojčica da mazi, **31.** ti ćeš da predeš, a ja? Ko će mene da mazi, a? **32.** Gde si video i čuo da miševi predu i da **33.** se umiljavaju!? Ko još mazi miševe?" **34.** "Možda si u pravu, a - možda i nisi." **35.** reče zamišljeno Moni. Jasna uze Monija **36.** u naručje i ode u svoju sobu. **37.** Tata i mama ostadoše da popiju kafu. **38.** Boni htede da pođe za Monijem ali odustade. **39.** Ode u kuhinju, šćućuri se iza šporeta i zadrema. **40.** Jasna je u svojoj sobi mazila Monija, **41.** a zatim uze sveske i knjige da radi **42.** domaće zadatke iz matematike i da uči. **43.** Moniju je bilo dosadno. Reši da **44.** malo odspava. Ali nije mogao. **45.** Razmišljao je o Boniju i njegovim rečima. **46.** Jadni Boni. Njega u ovoj kući niko **47.** neće voleti. Eto, on, Moni, je Jasnin **48.** kućni ljubimac. Onda mu sinu: a zašto **49.** Boni ne bi mogao da bude njegov **50.** kućni ljubimac?! Mama je otišla kod **51.** frizera, tata kod prijatelja na partiju šaha, **52.** a Jasna u školu. Moni siđe u kuhinju **53.** i tiho pozva Bonija. Boni izađe **54.** pitajući se u sebi da Moni nije promenio **55.** jelovnik. "Boni, imam ideju: ti ćeš biti **56.** moj kućni ljubimac! Nije loše, šta kažeš, a?! **57.** Šta ti je, Boni? " Boni je zateturao, **58.** zakolutao očima, ali se, najzad malo smiri. **59.** "Ma, ništa, u redu je. To, to ti misliš ozbiljno?! **60.** Vežeš mi lančić oko vrata i pokazuješ me prijateljima! "

Dutch – Musical Rain Condition

De Chinese Nachtegaal

Block 1
1. Het is nu heel lang geleden, maar **2.** daarom is het juist de moeite waard **3.** om het verhaal te horen, voor het in **4.** vergetelheid raakt. Het paleis van **5.** de Chinese keizer was het mooiste van **6.** de wereld, helemaal van fijn porselein, **7.** heel kostbaar, maar zo breekbaar **8.** en zo gevaarlijk om aan te raken dat **9.** je verschrikkelijk op moest passen. **10.** In de tuin zag je de wonderlijkste **11.** bloemen en aan de allermooiste **12.** waren zilveren belletjes gebonden, **13.** zodat je er niet voorbij kon gaan **14.** zonder de bloem te zien. Alles was **15.** heel geraffineerd in de tuin van de **16.** keizer en hij was zo uitgestrekt dat **17.** zelfs de tuinman niet wist waar hij ophield. **18.** Als je doorliep, kwam je in een **19.** heel mooi bos met hoge bomen **20.** en diepe meren. Dat bos liep tot **21.** aan de zee, die blauw en diep was; **22.** grote schepen konden zo onder de **23.** takken door varen. In die takken **24.** woonde een nachtegaal die zo lieflijk **25.** zong dat zelfs de arme visser, die **26.** toch zoveel andere dingen te **27.** doen had, stil bleef liggen om **28.** te luisteren, als hij 's nachts zijn **29.** netten binnenhaalde en dan de **30.** nachtegaal hoorde. "Lieve hemel, **31.** wat is dat mooi!" zei hij. Dan moest **32.** hij weer aan het werk en vergat hij **33.** de vogel; maar als die de volgende **34.** nacht weer zong en de visser weer **35.** op die plek was, dan zei hij hetzelfde: **36.** "Lieve hemel, wat is dat mooi!" **37.** Uit alle landen van de wereld **38.** kwamen er reizigers naar de stad **39.** van de keizer. Ze bewonderden **40.** de stad, het paleis en de tuin, **41.** maar als ze de nachtegaal hoorden, **42.** zeiden ze allemaal: "Dàt is het mooiste!" **43.** De reizigers vertelden erover als **44.** ze thuiskwamen en de geleerden **45.** schreven vele boeken over de stad, **46.** het paleis en de

tuin, maar ze vergaten **47.** de nachtegaal niet. Die stond bovenaan. **48.** En de mensen die gedichten konden **49.** schrijven, schreven de mooiste **50.** gedichten, allemaal over de nachtegaal **51.** in het bos aan de diepe zee. Die **52.** boeken kwamen overal ter wereld **53.** terecht en een paar ervan kwamen **54.** op een keer de keizer onder ogen. **55.** Hij zat in zijn gouden stoel en **56.** las en las, hij knikte telkens met **57.** zijn hoofd, want het deed hem **58.** genoegen om die prachtige **59.** beschrijvingen van de stad, het **60.** paleis en de tuin te horen.

Block 2

1. "Maar de nachtegaal is toch **2.** het allermooiste!" stond er geschreven. **3.** "Wat krijgen we nou?" zei de **4.** keizer. "De nachtegaal? Die ken **5.** ik helemaal niet! Is er zo'n vogel **6.** in mijn keizerrijk, en dan nog wel **7.** in mijn tuin? Dat heb ik nooit gehoord! **8.** Zoiets moet je dan uit een boek vernemen!" **9.** Toen riep hij zijn hofmaarschalk, die **10.** zo deftig was dat als iemand **11.** die lager in rang was dan hij, **12.** het waagde hem aan te spreken **13.** of hem iets te vragen, hij alleen **14.** maar "P!" zei en dat betekent niets. **15.** "Er moet hier een hoogst merkwaardige **16.** vogel zijn, die nachtegaal wordt **17.** genoemd," zei de keizer. "Men zegt **18.** dat dit het allermooiste in mijn **19.** grote rijk is! Waarom heeft **20.** niemand me dat verteld?" **21.** "Ik heb hem nog nooit eerder horen **22.** noemen," zei de hofmaarschalk. **23.** "Hij is nooit aan het hof voorgesteld!" **24.** "Ik wil dat hij hier vanavond komt zingen," **25.** zei de keizer. "De hele wereld weet wat **26.** ik heb en ik weet het zelf niet!" **27.** "Ik heb hem nog nooit horen noemen!" **28.** zei de hofmaarschalk. "Ik zal **29.** hem zoeken, ik zal hem vinden!" **30.** Maar waar was hij te vinden? **31.** De hofmaarschalk liep alle **32.** trappen op en af, zalen en **33.** gangen door; niemand van degenen **34.** die hij tegenkwam, had van de **35.** nachtegaal gehoord en de hofmaarschalk **36.** ging weer naar de keizer en zei **37.** dat het waarschijnlijk een fabeltje **38.** was van de mensen die boeken schreven. **39.** "Uwe keizerlijke Majesteit moet niet geloven **40.** wat er geschreven wordt. Dat zijn **41.** verzinsels en dat is wat ze zwarte **42.** kunst noemen!" "Maar het boek **43.** waar ik het in gelezen heb," zei **44.** de keizer, "is me door de verheven **45.** keizer van Japan gestuurd en dan **46.** kan het geen onwaarheid bevatten. Ik **47.** wil de nachtegaal horen! Vanavond moet **48.** hij hier zijn! Hij heeft mijn hoogste gunst! **49.** En komt hij niet, dan wordt het hele **50.** hof na het avondeten op de buik gestompt." **51.** " Tsing-pe!" zei de hofmaarschalk **52.** en hij rende weer alle trappen op en af **53.** en alle gangen en zalen door; **54.** en het halve hof liep mee, **55.** want ze wilden niet zo graag op **56.** hun buik worden gestompt. **57.** Het was me een gevraag naar **58.** die merkwaardige nachtegaal, **59.** die de hele wereld kende, **60.** maar niemand aan het hof.

Comprehension Questions

***Block 1:** Het paleis van de Chinese keizer was het mooiste van de wereld, helemaal van goud en heel kostbaar. (N); Het paleis was zo breekbaar en zo gevaarlijk om aan te raken dat je verschrikkelijk moest oppassen. (Y); Aan de allermooiste bloemen in de tuin waren gouden belletjes gebonden. (N); De tuin was zo uitgestrekt dat zelfs de tuinman niet wist waar hij ophield. (Y); Als je de tuin doorliep kwam je bij een heel mooi bos dat liep tot aan de zee. (Y); In de takken van het bos woonde een nachtegaal. (Y); De nachtegaal zong zo lieflijk dat zelfs de arme houthakker bleef staan om te luisteren. (N); Reizigers uit de hele wereld bewonderden de stad, het paleis en de tuin, maar vonden de nachtegaal het mooist. (Y); Mensen die gedichten konden schrijven, schreven allemaal over de nachtegaal in het bos aan de diepe zee (Y); Het deed de keizer geen genoegen om die prachtige beschrijvingen van de stad, het paleis en de tuin te horen. (N)*

***Block 2:** De Chinese keizer had nog nooit van de nachtegaal uit zijn tuin gehoord. (Y); Iedereen lager in rang dan de hofmaarschalk durfde hem aan te spreken. (N); De keizer wil niet dat de nachtegaal hier vanavond komt zingen. (N); De hofmaarschalk ging de nachtegaal zoeken. (Y); Iedereen die de hofmaarschalk tegenkwam had de nachtegaal gehoord. (N); De hofmaarschalk zei tegen de keizer dat het verhaal over de nachtegaal een fabeltje was van de mensen die de boeken schreven. (Y); Het boek waarin de keizer gelezen heeft over de nachtegaal is door de keizer van Mongolië gestuurd. (N); Als de nachtegaal niet zou komen dan zou het hele hof na het avondeten op het hoofd worden gestompt. (N); Het halve hof hielp mee zoeken, want ze wilden niet zo graag op hun buik worden gestompt. (Y); Iedereen aan het hof kende de nachtegaal. (N)*

De Feeën

Block 1

1. Er was eens een weduwe en die had **2.** twee dochters. De oudste leek sprekend **3.** op haar, zowel qua uiterlijk als qua **4.** karakter. Als je haar zag zou je zweren **5.** dat het de moeder was. Want ze waren **6.** allebei zo gemeen en trots dat het **7.** niet om uit te houden was. De jongste **8.** leek precies op haar vader, zo vriendelijk **9.** en aardig als ze was, en bovendien was zij **10.** een van de mooiste meisjes die men van **11.** heinde en verre kon vinden. Omdat **12.** men gewoonlijk het meest houdt van **13.** iemand die op hem lijkt, was de moeder **14.** dol op haar oudste dochter en had zij **15.** tegelijk een verschrikkelijke hekel aan **16.** de jongste. Die moest in de keuken eten, **17.** en altijd maar werken. Onder andere **18.** moest het arme kind tweemaal per **19.** dag water gaan halen bij de bron, ruim **20.** een halve mijl van het huis, en met **21.** een grote kruik vol terugkomen. **22.** Op een dag, toen zij weer bij die **23.** bron was, kwam er een arme vrouw **24.** naar haar toe en vroeg of zij haar **25.** te drinken wilde geven. "Natuurlijk, **26.** moedertje," zei het mooie meisje. **27.** Zij schepte toen water uit de helderste **28.** plek van de bron en reikte het haar **29.** aan, terwijl ze aldoor de kruik vasthield, **30.** zodat de vrouw makkelijker kon drinken. **31.** Toen de goede vrouw gedronken had, **32.** sprak zij tegen haar: "Je bent zo mooi **33.** en goed en vriendelijk, dat ik zin heb **34.** om je een geschenk te geven

(want zij **35.** was een fee die de gedaante van een **36.** arme boerenvrouw had aangenomen, **37.** om te zien hoe ver de vriendelijkheid **38.** van het meisje wel zou gaan). "Ik geef **39.** je als geschenk," zei de fee, "dat er bij **40.** ieder woord dat je zegt een bloem **41.** of een edelsteen uit je mond valt." **42.** Toen het mooie meisje thuiskwam **43.** schold haar moeder haar uit, dat ze **44.** zo lang bij de bron was gebleven. **45.** "Vergeef mij, moeder, dat ik zo laat ben." **46.** En terwijl ze dat zei vielen er twee rozen, **47.** twee parels en twee grote diamanten **48.** uit haar mond. "Wat is dat?" riep haar **49.** moeder, één en al verbazing. "Ik **50.** geloof dat er uit haar mond parels **51.** en diamanten rollen! Hoe komt dat , **52.** m'n kind?" Het was de eerste keer **53.** dat zij haar m'n kind noemde. **54.** Argeloos vertelde het arme meisje alles **55.** wat haar overkomen was, en onderwijl **56.** viel er een eindeloze hoeveelheid diamanten **57.** uit haar mond. "Daar moet ik toch **58.** werkelijk mijn oudste dochter ook eens **59.** heen sturen," zei de moeder. "Zeg Fanchon, **60.** kijk eens wat er uit de mond van je

Block 2
1. zuster komt, als ze gaat praten; zou **2.** je het niet heerlijk vinden als je ook die **3.** gave had? Je hoeft er niets anders **4.** voor te doen dan water te gaan halen **5.** bij de bron en als er dan een arme vrouw **6.** komt die je te drinken vraagt dan moet **7.** je het haar vriendelijk geven." "Nu nog **8.** mooier," antwoordde het brutale meisje, **9.** "ik zie mij al naar de bron gaan!" "Ik wil **10.** dat je er heen gaat!" hernam de moeder. **11.** "Onmiddellijk!" Toen moest zij wel. **12.** Zij ging op weg, maar mopperde aan **13.** één stuk door. Zij nam de mooiste zilveren **14.** fles mee, die er in huis was. Amper was **15.** zij bij de bron aangekomen of ze zag **16.** een prachtig geklede dame uit het bos **17.** te voorschijn treden, die haar te drinken **18.** vroeg. Het was dezelfde fee die aan haar **19.** zuster verschenen was, maar nu had zij **20.** de gedaante en de kleding van een prinses **21.** aangenomen, om te zien hoe ver de **22.** onvriendelijkheid van dat meisje wel zou **23.** gaan. "Dacht u soms," zei die trotse en **24.** brutale meid, "dat ik hier gekomen ben **25.** om u te drinken te geven? Wel ja, die zilveren **26.** fles heb ik natuurlijk expres meegebracht **27.** om er Mevrouw uit te laten drinken; **28.** dat kunt u net denken! Drink maar **29.** uit uw hand, als u zo'n dorst hebt." **30.** "Je bent niet erg vriendelijk," sprak de fee, **31.** zonder zich boos te maken. "Goed dan, **32.** omdat je zo weinig behulpzaam bent zal ik **33.** jou ook een geschenk geven: bij ieder woord **34.** dat je zegt zal er een slang of een pad **35.** uit je mond komen." Zodra de moeder **36.** haar zag, riep zij haar toe: "Hoe ging het **37.** m'n dochter?" "Hoe ging het?.. moeder!" **38.** antwoordde het brutale nest, en meteen **39.** vielen er twee slangen en twee padden **40.** uit haar mond. "Grote hemel!" riep de **41.** moeder, "wat zie ik nou? Dat is de schuld **42.** van je zuster, dat zal ik haar betaald zetten!" **43.** En meteen stoof ze op haar af om haar **44.** een pak slaag te geven. Het arme kind **45.** rende weg en vluchtte naar het bos, **46.** daar vlakbij. De zoon van de koning, **47.** die juist van de jacht terugkeerde, **48.** kwam haar tegen en toen hij zag hoe **49.** mooi zij was vroeg hij wat zij daar deed, **50.** zo moederziel alleen, en waarom zij **51.** huilde. "Ach, mijnheer, mijn moeder **52.** heeft mij het huis uitgejaagd!" De zoon **53.** van de koning zag toen vijf of zes parels **54.** en evenveel diamanten uit haar mond **55.** rollen en vroeg haar hoe dat kwam. Zij **56.** vertelde hem alles wat haar overkomen was. **57.** Daardoor werd de zoon van de koning verliefd **58.** op haar en nam haar mee naar het paleis **59.** van zijn vader. Hij vond dat zo'n gave meer **60.** waard was dan alles wat een andere vrouw

Comprehension Questions

Block 1: *Het verhaal gaat over een weduwe die twee dochters had. (Y); De oudste dochter leek sprekend op haar vader, zowel qua uiterlijk als qua karakter. (N); De jongste dochter leek precies op haar moeder, zo vriendelijk, aardig en mooi als ze was. (N); De moeder was dol op haar oudste dochter en had een verschrikkelijke hekel aan de jongste dochter. (Y); Toen het mooie meisje een keer bij de bron was, wilde zij een arme vrouw niet te drinken geven. (N); De arme vrouw was een fee die de gedaante van een arme vissersvrouw had aangenomen. (N); De arme vrouw gaf het mooie meisje een geschenk. (Y); Toen het mooie meisje thuiskwam vroeg haar moeder vriendelijk waarom ze zo lang was weggebleven. (N); Uit de mond van de jongste dochter vielen drie rozen, drie parels en drie grote diamanten. (N); De moeder zei dat ze haar oudste dochter toch ook eens naar de bron moest sturen. (Y)*

Block 2: *Het oudste meisje ging op weg naar de bron, maar mopperde aan één stuk door. (Y); Een sjofel geklede dame kwam uit het bos tevoorschijn en vroeg het oudste meisje te drinken. (N); De fee had de gedaante en de kleding van een prinses aangenomen. (Y); Het oudste meisje zei dat de dame maar uit haar hand moest drinken als ze zo'n dorst heeft. (Y); Het geschenk voor de oudste dochter was dat er bij ieder woord dat ze zou zeggen een slang of een pad uit haar mond zou komen. (Y); De moeder zei dat dit haar eigen schuld was. (N); Het oudste meisje rende weg en vluchtte naar het bos daar vlakbij. (N); De zoon van de koning kwam het jongste meisje tegen en zag hoe mooi zij was. (Y); De zoon van de koning zag vijf of zes parels en evenveel diamanten uit de mond van het jongste meisje vallen. (Y); De zoon van de koning werd verliefd op de oudste dochter en nam haar mee naar het paleis van zijn vader. (N)*
